# Growing nickel supply from the tropics threatens priority conservation areas

**DOI:** 10.1038/s41559-026-03068-4

**Published:** 2026-05-06

**Authors:** Jayden Hyman, Laura J. Sonter, Eve McDonald-Madden, James E. M. Watson, Evelyn M. Mervine, Joseph W. Bull, Chloe Dawson, Thomas J. Lloyd, Sebastian Luckeneder, Martine Maron, Bernardo Mendonca Severiano, Sarah Raymond, Thomas A. Schlacher, Rachakonda Sreekar, Rick K. Valenta, Piero Visconti, Tim T. Werner, Stephen A. Northey

**Affiliations:** 1 School of the Environment, The University of Queensland, Brisbane, Queensland Australia; 2 Centre for Biodiversity and Conservation Science, The University of Queensland, Brisbane, Queensland Australia; 3 Sustainable Minerals Institute, The University of Queensland, Brisbane, Queensland Australia; 4 The Biodiversity Consultancy, Cambridge, UK; 5 Department of Biology, University of Oxford, Oxford, UK; 6 College of Science & Engineering, James Cook University, Townsville, Queensland Australia; 7 Stockholm Resilience Centre, Stockholm University, Stockholm, Sweden; 8 Institute for Sustainable Futures, University of Technology Sydney, Ultimo, New South Wales Australia; 9 School of Science, Technology and Engineering, University of the Sunshine Coast, Maroochydore, Queensland Australia; 10 Biodiversity, Ecology, and Conservation Group, Biodiversity and Natural Resources Program, International Institute for Applied Systems Analysis, Laxenburg, Austria; 11 School of Geography, Earth and Atmospheric Sciences, The University of Melbourne, Carlton, Victoria Australia

**Keywords:** Environmental impact, Sustainability, Biodiversity

## Abstract

Increasing global demand for nickel, an essential metal in low-carbon technologies and stainless steel, is driving a surge in mining in strongholds of tropical biodiversity. We use a global mine-by-mine supply scenario model to quantify the trade-off between meeting future nickel demand for decarbonization and conserving areas critical for achieving biodiversity and climate targets. Nickel laterites—near-surface deposits often found beneath tropical forests—account for 78 to 83% of modelled supply between 2025 and 2050. Over this timeframe, half of mined nickel threatens the top 10% of global land areas most critical for conserving biodiversity and storing carbon, but avoiding mining in these areas increases the risk of supply shortfalls. In addition, 53 to 60% of future supply comes from coastal mines, which threaten the top 10% of global priority areas for conserving marine biodiversity. While deep-sea resource development remains controversial, we show that a moratorium may increase reliance on nickel sourced from high-priority areas for conserving terrestrial and coastal marine biodiversity. Securing ecologically responsible nickel supply requires integrating terrestrial and marine conservation priorities to inform sourcing and mine development decisions, alongside efforts to mitigate unavoidable impacts, increase resource exploration and reduce long-term demand.

## Abstract (web summary)

Scenario modelling of nickel supply to meet demand to 2050 identifies extensive overlap between land and coastal mines and high-priority areas for terrestrial and marine conservation, and shows how this tension would increase under a moratorium on deep-sea mining.

## Main

The global transition to renewable energy is driving unprecedented demand for mined metals^1–3^. Due to their widespread use in low-carbon technologies, copper and nickel require the most mining in terms of total ore extraction compared to other energy transition metals^4^. Metal mining in the tropics has been rising, causing widespread deforestation in countries such as Indonesia, the Philippines and Brazil^5^. The biggest increases in nickel mining have been in Indonesia^6^, where production has increased tenfold in the past decade (supported by foreign investment from China), now reaching more than half of global supply^7^. This surge in nickel mining in Indonesia will probably continue and could reach as high as 74% of global supply by 2040^3^. The rapid increase in supply from tropical sources has led to a decline in the nickel price that has driven the closure of mines in other jurisdictions such as Australia^7^.

The projected increase in nickel mining over the next two decades will present critical social and environmental trade-offs^8–11^. Recent studies show that the land-use intensity per tonne of nickel mined in Indonesia is 20 times higher than industry estimates^12^ and ranges from 4 to 500 times higher for individual nickel mines worldwide, potentially causing irreplaceable biodiversity loss and substantial carbon emissions from under-accounted deforestation^13^. While mining can be associated with improved local living standards for some communities, the impacts of deforestation and pollution can undermine these benefits over time, as shown by declines in overall well-being and increasing conflict near nickel mines in Indonesia^14,15^. Moreover, nickel processing has remarkably high carbon emissions, owing to the energy-intensive processes required for smelting and refining laterite ores, combined with the use of captive coal-fired power stations^7,15,16^. Securing ecologically responsible nickel supply for decarbonization is increasingly urgent to avoid undermining conservation and climate change mitigation goals.

Global conservation targets, including commitments to protect at least 30% of land and ocean areas by 2030, could influence nickel sourcing and mine permitting decisions if key deposits are located in priority areas that are irreplaceable for protecting biodiversity and storing carbon^13,17^. At the same time, efforts to address risks arising from the geographic concentration of supply are increasing (including a proposal for a Global Minerals Trust^18^), as are investments in research and development of novel resource streams^2^. For instance, deep-sea mining has recently entered mainstream discourse following the United States Executive Order Unleashing America’s Offshore Critical Minerals and Resources, and the first commercial operations could begin within years, potentially providing an unconventional source of nickel^3,19^. However, deep-sea mining currently faces substantial opposition, with several countries, businesses and non-governmental organizations calling for a moratorium due to concerns of potential impacts to remote, relatively intact and often poorly understood deep-sea ecosystems^20–23^. To date, the potential implications of global conservation actions for nickel supply—such as avoiding mining in priority conservation areas or implementing a moratorium on deep-sea mining—remain underexplored and are a key knowledge gap for the nickel sector^24^.

**Extended Data Fig. 1:**
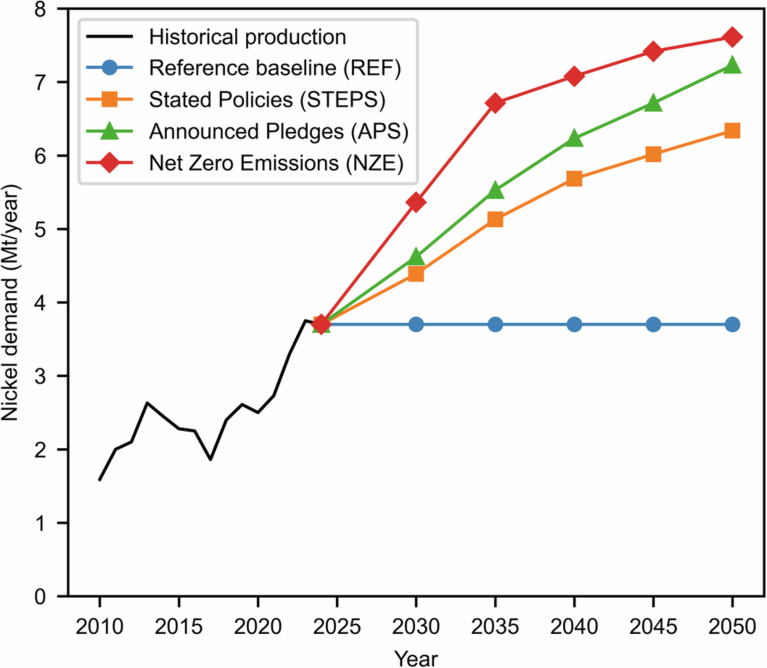
Historical nickel production from 2010 to 2024^71^ and predicted nickel demand under a range of scenarios to 2050. We provide a baseline reference scenario of no growth in nickel demand beyond 2024 production of 3.7 Mt. All other demand scenarios are from the International Energy Agency’s Global Critical Minerals Outlook 2025^3^.

**Extended Data Fig. 2:**
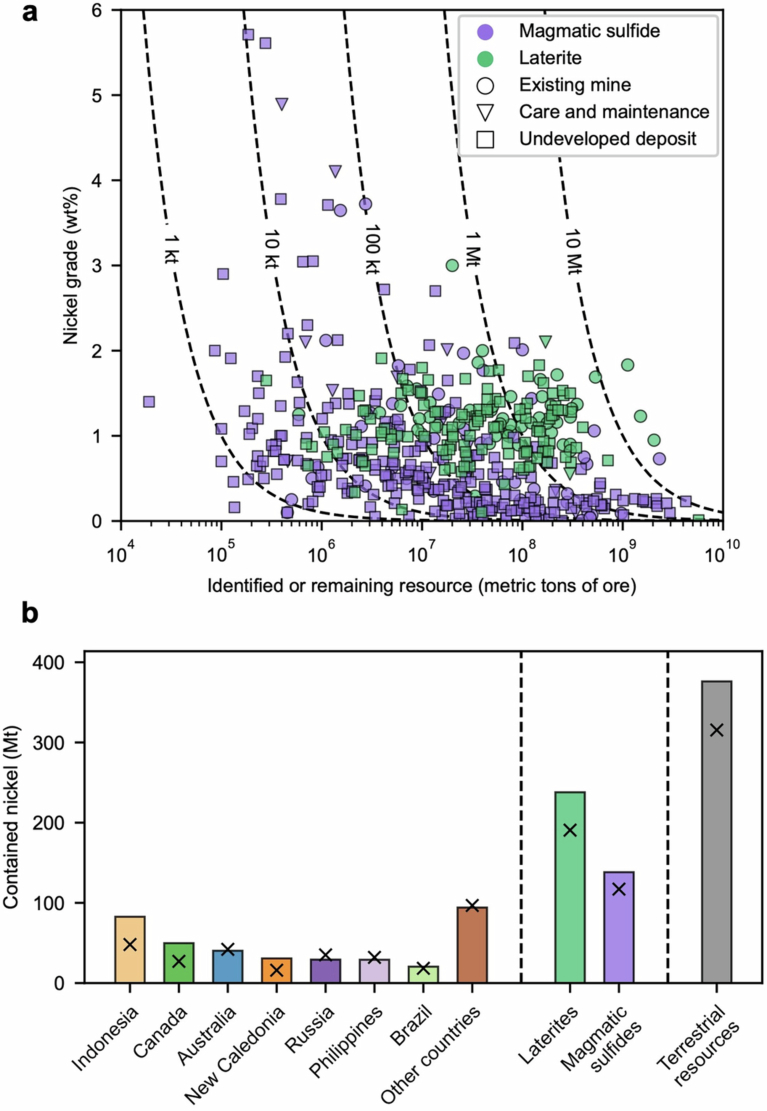
Global terrestrial nickel resources. **a**, Resource tonnage and grade of global nickel deposits, including status for the year 2024. The dataset includes 487 projects, with 179 laterite deposits and 308 magmatic sulfide deposits. The project dataset was compiled from recent studies^13,26^, with deposit characteristics (ore grade and tonnage) sourced from the S&P Capital IQ Pro Database, capturing reported resources (including reserves) to the year 2025. Deposit ore grade and tonnage, together with other factors like processing and infrastructure costs, play an important role in evaluating the economic viability of mining projects^62^. **b**, Nickel contained in terrestrial deposits by country and deposit type. Bars show estimates from this study for the year 2025, and crosses show estimates from Mervine et al.^13^ for the year 2020.

**Extended Data Fig. 3:**
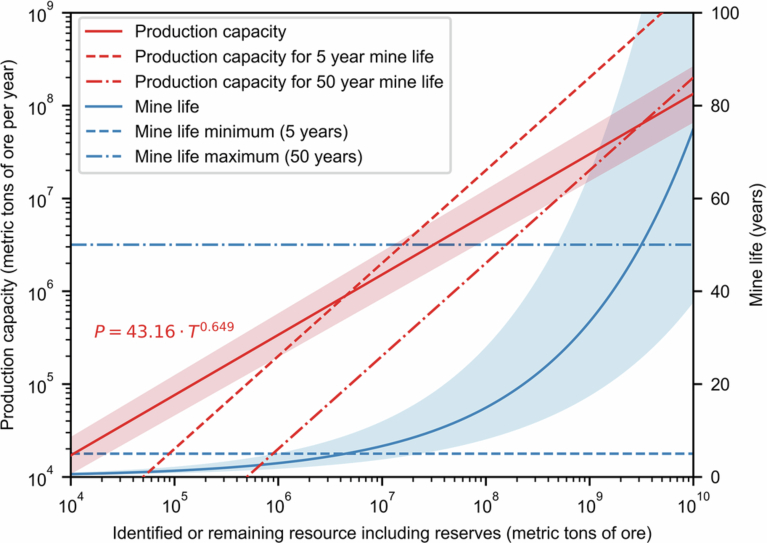
Relationship between ore production capacity, resource size, and mine life. Taylor’s rule was used to estimate future production for existing and new mines using a re-estimation of the rule for open-pit plus underground (block cave) mines^61^, adjusted for annual production at 350 operating days per year. Solid lines show the estimated ore production capacity and corresponding mine life from the regression model of Long et al.^61^, and shaded bands show the range derived from the upper and lower 95% confidence interval bounds of the regression coefficients. Dotted lines show an upper and lower cut-off in production capacity constrained by a minimum mine life of 5 years and a maximum mine life of 50 years, respectively.

In this study, we model whether known nickel resources can meet growing demand and quantify how conservation policies could reshape global supply. We present a global implementation of the Primary Exploration, Mining and Metal Supply Scenario (PEMMSS) model^25^ to simulate mine-by-mine development, production and depletion of global nickel resources under the International Energy Agency’s demand projections (Extended Data Fig. 1). The PEMMSS model uses a global supply–demand balance algorithm to translate commodity demand into stochastic mine development scenarios, preserving path dependencies and deposit-level spatial resolution (Methods). We compile a comprehensive dataset of nickel mines and undeveloped deposits (Extended Data Fig. 2) from recent studies^13,26^ and the S&P Capital IQ Pro Metals and Mining Database^27^ and map these deposits in the context of global priority areas for conserving biodiversity and storing carbon^17,28^. These areas have been identified as essential to protect if we are to minimize the number of future extinctions of plant and animal species. Our modelling explores regional supply from existing mines and known deposits between 2025 and 2050, including whether global resource estimates are sufficient to meet projected demand through a stochastic simulation of mine production capacity (Extended Data Fig. 3). We run multiple scenarios that reflect potential global conservation policies and associated actions (Extended Data Table 1). First, we exclude mining projects in priority conservation areas for protecting biodiversity and carbon (the top 0 to 30% in 5% increments) to explore whether demand can be met from known resources while minimizing the amount of mining within these priority areas. Second, we increase delays to the potential start of deep-sea mining (0 to 20-year delay in 5-year increments) consistent with different policies supporting a moratorium. Our analysis identifies a critical trade-off between ambitions to secure future nickel supply for decarbonization and the global conservation actions required for ecologically responsible mining.

## Results and discussion

### Nickel supply is shifting to the tropics

**Fig. 1:**
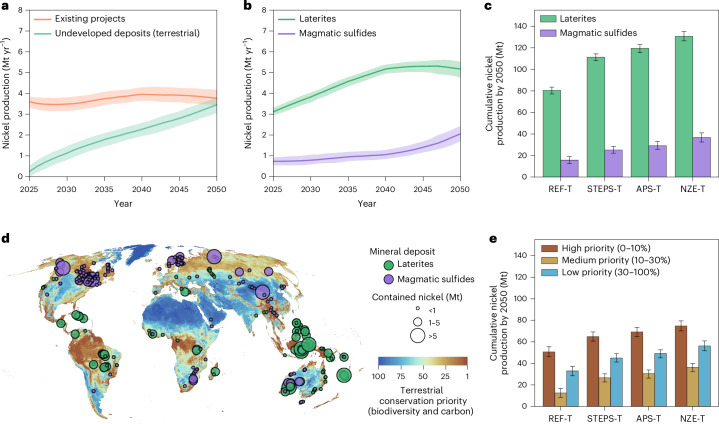
Global nickel supply from known terrestrial resources under future demand scenarios between 2025 and 2050. **a**–**c**,**e**, Nickel production is categorized by initial project status (**a**), deposit type (**b**,**c**) and priority areas for biodiversity and carbon (**e**). **d**, The global map shows the location and estimated nickel contained within known deposits overlaid onto terrestrial conservation priorities for conserving biodiversity and storing carbon^17^. The line charts in **a** and **b** show the median annual terrestrial nickel production under the Announced Pledges Scenario (APS-T, where T denotes terrestrial resources only) across model simulations (*n* = 500) at each timestep, and the shaded bands show the 5th to 95th percentile range. The column charts in **c** and **e** show the median cumulative nickel production across model simulations (*n* = 500) between 2025 and 2050, and the error bars show the 5th and 95th percentiles. Cumulative terrestrial production estimates vary across demand scenarios in **c** and **e**, including the baseline reference scenario of no demand growth (REF-T), the Stated Policies Scenario (STEPS-T), the Announced Pledges Scenario (APS-T) and the Net Zero Emissions by 2050 Scenario (NZE-T). Data for **d** from ref. ^17^.

**Extended Data Fig. 4:**
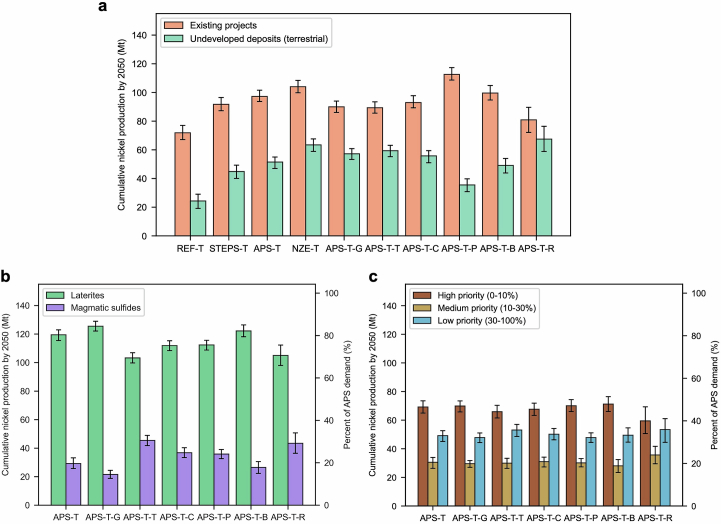
Sensitivity of cumulative terrestrial nickel production between 2025 and 2050 to alternative demand scenarios and value models. **a**, Sensitivity of cumulative nickel production from existing projects and undeveloped deposits to a baseline reference scenario (REF-T), three demand scenarios from the International Energy Agency (Extended Data Fig. 1), and six alternative value models (Extended Data Table 1). **b**–**c**, Across value models, we show the percent of demand met by each deposit type (**b**), and from high, medium and low priority conservation areas on land (**c**) under the Announced Pledges Scenario from terrestrial resources (APS-T). Values show median cumulative production across model simulations (n = 500) between 2025 and 2050, and the error bars show the 5th and 95th percentiles.

**Extended Data Fig. 5:**
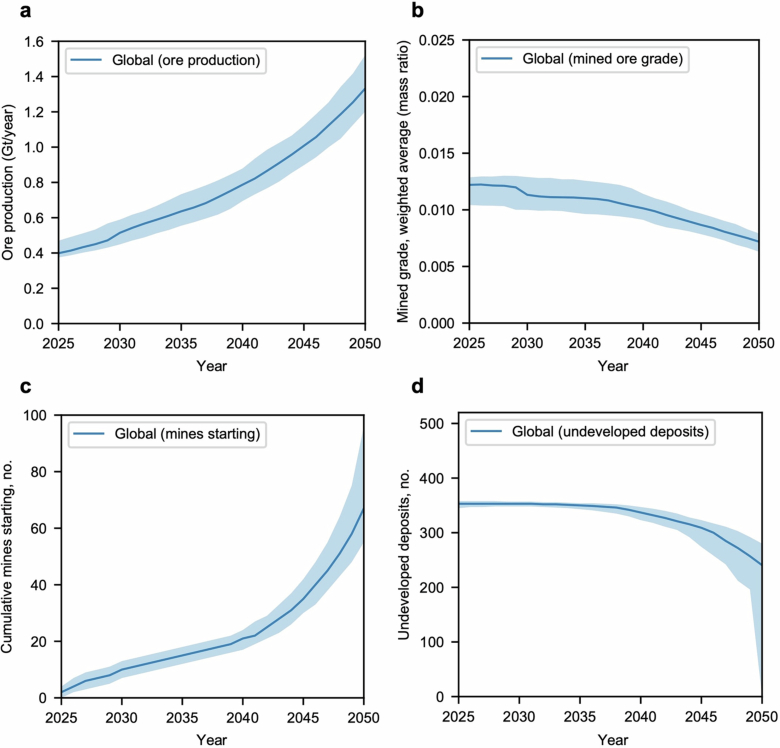
Global terrestrial ore production, mined ore grade, mines starting and undeveloped deposits under the Announced Pledges Scenario (APS-T). **a**–**d**, The line charts show the median values across model simulations (n = 500) for global terrestrial ore production (**a**), mined ore grade (**b**), the cumulative number of mines starting production (**c**), and the remaining undeveloped deposits (**d**). The shaded bands show the 5th to 95th percentile range.

On the basis of current global resource and reserve estimates, our model suggests that existing projects (that is, those having commenced production before 2025) can maintain between 3.2 and 4.3 Mt of nickel production per year until 2050 under scenarios where there are no constraints on exploitation of known resources at these sites (Fig. 1a). However, our scenario modelling shows that supply from existing mines alone may be insufficient to meet future nickel demand, as estimated by the International Energy Agency (Extended Data Fig. 4a). Under the Announced Pledges Scenario (reflecting global demand to meet decarbonization targets), a 43% increase in annual nickel production is required over the next decade, which could be delivered by rapidly developing identified terrestrial resources (an additional 1.5 to 2.1 Mt of nickel mined per year by 2035), again assuming no constraints on exploitation of these sites (Fig. 1a). Under this scenario, our modelling shows a potential threefold increase in annual ore production, a decline in average mined ore grade to below 1% and an estimated 55 to 96 new nickel mines starting production by 2050 (Extended Data Fig. 5).

Secondary supply (nickel recovered from recycling) will become increasingly important by mid-century to reduce reliance on mined nickel but is projected to play a minor role in the near-term (~2% in 2024, ~1.5–3% by 2030 and ~5–10% by 2040 across the Stated Policies and Announced Pledges scenarios^3,29^), constrained by the rate at which products reach their end of life for recycling^3^. Nickel is predominantly used in long-lived applications, including stainless steel in buildings and infrastructure (~50-year lifespan) and industrial machinery (~25 years) (refs. ^30,31^). The amount of nickel available for recycling today reflects the lower production volumes of past decades^26^, and the in-use stocks being accumulated now may only become available for recycling at meaningful volumes by mid-century^3,30,32^. Current end-of-life recycling rates fall far short of what is needed to offset primary supply requirements, and scaling secondary supply will require improvements in collection systems, urban mining and design for recyclability^32,33^. Even with increasing adoption of low- or no-nickel battery chemistries^3,7^, total demand is projected to grow substantially across all of the International Energy Agency’s scenarios due to nickel’s importance for stainless steel, renewables and other applications (Extended Data Fig. 1).

**Extended Data Fig. 6:**
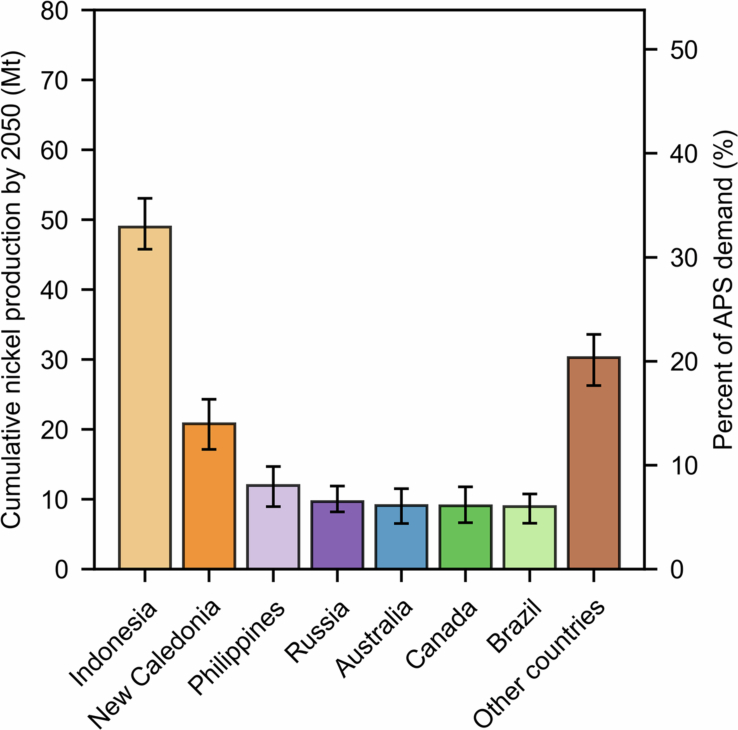
Cumulative terrestrial nickel production by country under the Announced Pledges Scenario (APS-T). Values show median cumulative production across simulations (n = 500) between 2025 and 2050, and the error bars show the 5th and 95th percentiles.

Global terrestrial nickel resources can be broadly classified as either laterite or magmatic sulfide deposits (Fig. 1), and the environmental impacts of mining can vary substantially by deposit and country^13,26,34^. We find that all supply from the top three producing countries—Indonesia, New Caledonia and the Philippines—comes from surficial laterite ores (Fig. 1d). These ore bodies form through weathering of near-surface ultramafic rocks often beneath tropical forest ecosystems and require progressive clearing and strip mining throughout the life of a project^12^. Most large (>1 Mt Ni) laterite deposits occur in the tropics with relatively high biomass carbon (Fig. 1d), although some occur in modern-day arid climates (for example, Western Australia), having formed under past tropical conditions^13^. In contrast, magmatic sulfide deposits originate from deeper magmatic processes that concentrate nickel in sulfide mineral ore bodies that are extracted in underground mines and open pits (often with lower surficial footprints), with major deposits occurring at higher latitudes in Australia, Canada and Russia (Fig. 1d). Our modelling shows a continued increase in nickel supply from deposits in the tropics over the next decade (Fig. 1b), where laterites produce 116 to 123 Mt of nickel between 2025 and 2050 (78 to 83% share of demand) under the Announced Pledges Scenario—approximately four times more nickel than from magmatic sulfides across demand scenarios (Fig. 1c). This surge in mining in the tropics creates a complex trade-off for decarbonization due to the relatively high carbon dioxide equivalent (CO_2_e) emissions from strip mining (0.043 to 5.55 t CO_2_e t^−1^ Ni) and processing (18 to 120 t CO_2_e t^−1^ Ni) of laterite ore bodies^7,13^. Our results are consistent with recent shifts in regional production, with Indonesian laterites being the most competitive and largest contributor to future supply based on deposit characteristics (Extended Data Fig. 6 and Extended Data Fig. 4b).

Notably, several Australian nickel mines in our dataset (Mount Keith, Ravensthorpe, Savannah, Cosmos and Avebury) and the Koniambo mine in New Caledonia closed in 2024 and are now in an indefinite state of care and maintenance due to the rapid and unanticipated surge in lower-cost Indonesian nickel. Premature mine closure due to competition may lead to poor environmental outcomes, because much of the land-use impact occurs up front during construction and the first few years of production (particularly for deeper magmatic sulfide deposits), and the economic returns needed for effective mine rehabilitation may never materialize. A key question remains: can higher-cost producers, even those operating under potentially stricter environmental standards, compete in a market increasingly dominated by lower-cost supply from the tropics?

### Nickel supply threatens conservation priorities

Priority conservation areas represent the most important global land areas to protect from industrial human pressures to minimize the risk of extinction to plants and vertebrates and the loss of carbon stocks (biomass carbon and vulnerable soil carbon) and are, therefore, critical to achieving the goals of the biodiversity and climate conventions^17^. Our scenario modelling reveals that approximately half of nickel production between 2025 and 2050 could be sourced from mining projects located in the top 10% of priority areas for terrestrial biodiversity and carbon conservation (44 to 49% share of demand under the Announced Pledges Scenario; Fig. 1e). This finding remains relatively consistent (that is, plus or minus ~5% share of demand) when subject to a range of sensitivity analyses, including high and low demand projections (Fig. 1e), relative deposit value models and brownfield expansion scenarios (Extended Data Fig. 4c).

**Fig. 2:**
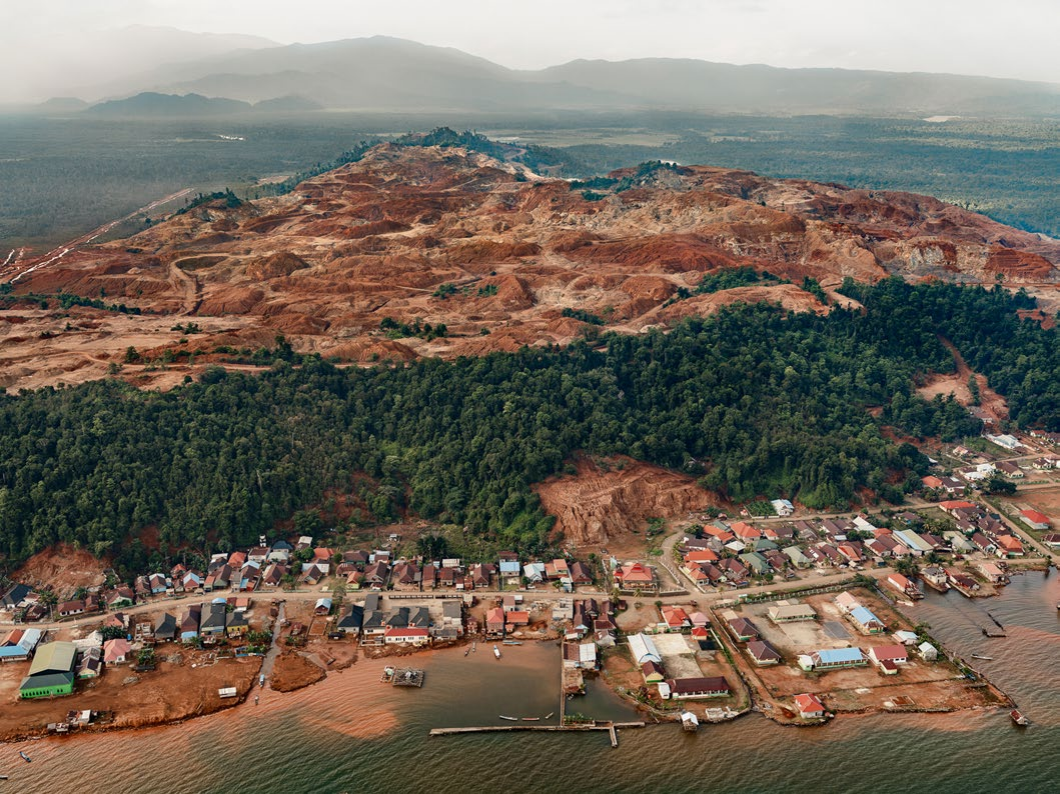
Coastal nickel laterite mining in Sulawesi, Indonesia. The aerial imagery depicts a mine site near remnant forest, a coastal village and sediment-laden coastal waters. Credit: Tom Hegen.

**Fig. 3:**
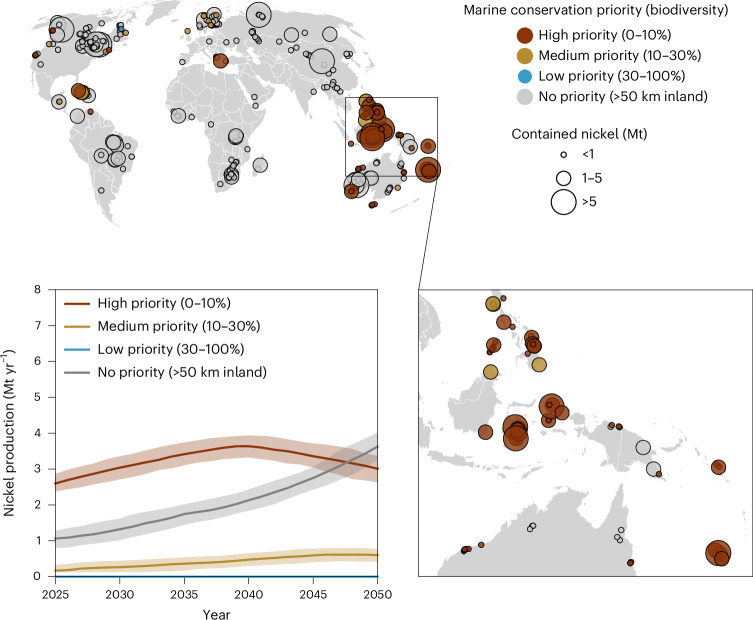
Terrestrial nickel mining threats to priority areas for conserving marine biodiversity. Mines located within 50 km of a coastline were categorized as threatening high-, medium- or low-priority marine areas based on the mean priority value of adjacent coastal waters, and inland mines (>50 km from coastline) were not assigned a priority value. Priority areas for conserving marine biodiversity are from Sala et al.^28^. The line chart shows the median annual terrestrial nickel production from projects under the Announced Pledges Scenario (APS-T) across model simulations (*n* = 500), categorized by the threat to priority areas for conserving marine biodiversity. Shaded bands show the 5th to 95th percentile range across simulations. Only one project was assigned as a threat to low-priority marine areas but did not materially contribute to supply under the APS-T. Data from ref. ^28^.

**Extended Data Fig. 7:**
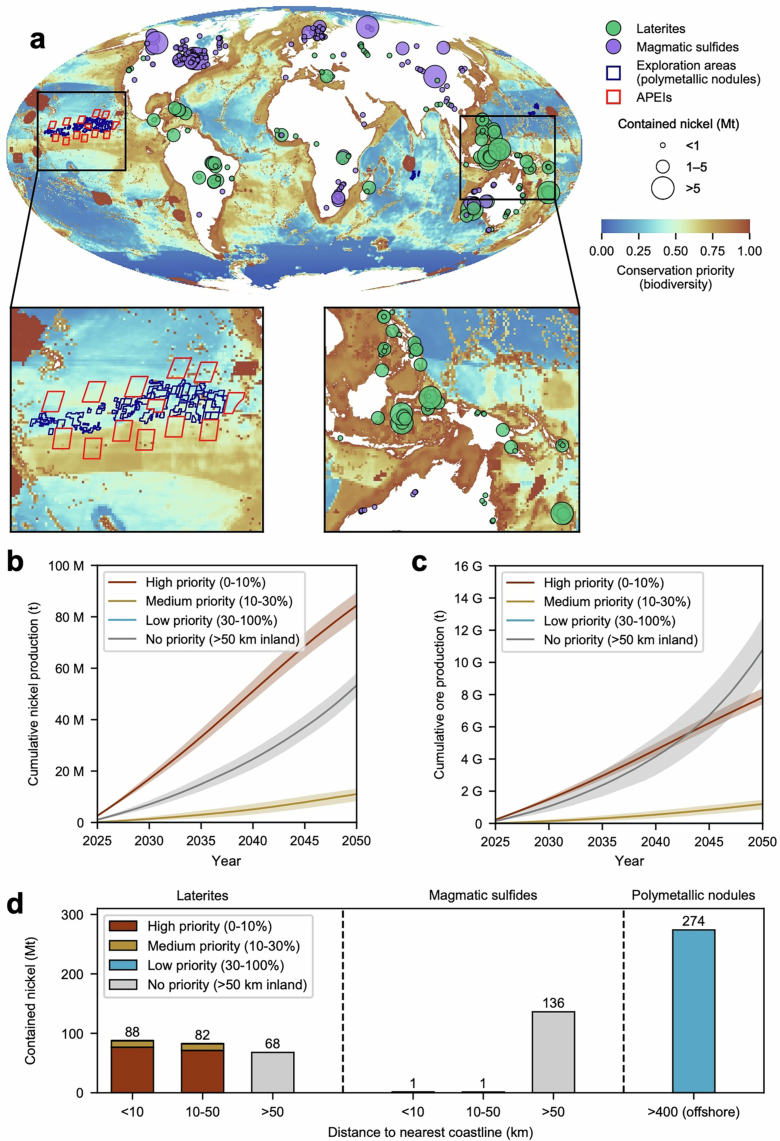
Cumulative nickel production under the Announced Pledges Scenario near priority areas for conserving marine biodiversity. The global map (**a**) shows terrestrial nickel deposits, as well as current exploration areas for polymetallic nodules and Areas of Particular Environmental Interest (APEIs) in the Clarion-Clipperton Zone, overlaid onto ocean biodiversity priorities^28^. The priority ranking reflects the most important areas to protect to conserve marine biodiversity based on several biodiversity features including species extinction risk, species ranges, evolutionary history and biogeographic representation^28^. We show median cumulative nickel (**b**) and ore (**c**) production for coastal mines within 50 km of high, medium and low priority areas for conserving marine biodiversity across model simulations (n = 500). Shaded bands show the 5th to 95th percentile range. Inland mines greater than 50 km from the nearest coastline are not assigned a priority rank. Nickel contained in deposits is categorized by distance to nearest coastline and priority rank (**d**). Data for **a** from ref. ^28^.

Several major terrestrial nickel laterite deposits also occur near areas of high conservation importance for marine biodiversity (Extended Data Fig. 7a), including on the Indonesian islands of Sulawesi (Fig. 2), Maluku and Raja Ampat in the Coral Triangle. An estimated 170 Mt of nickel contained in laterite deposits occurs within 50 km of coastlines (88 Mt Ni within 10 km; 82 Mt Ni between 10 and 50 km), compared to just 2 Mt for magmatic sulfide deposits, which are predominantly located greater than 50 km inland (Extended Data Fig. 7d). Our modelling shows that 53 to 60% (79 to 90 Mt) of nickel could be sourced from mines within 50 km of coastal waters which rank in the top 10% of global priority areas for conserving marine biodiversity (Fig. 3 and Extended Data Fig. 7b). This requires mining and processing an estimated 7.4 to 8.4 Gt of ore between 2025 and 2050 upstream of global strongholds of marine biodiversity, generating billions of tonnes of waste that would need to be deposited in tailings storage facilities (Extended Data Fig. 7c). Throughout these mining operations, and even following their closure, ongoing sediment and heavy metal contamination, the potential for catastrophic tailings storage facility failures and practices of marine or riverine tailings disposal, could pose substantial threats to coastal ecosystems, biodiversity, fisheries and local communities^26,35–37^. Our modelling reveals that 36 to 42% (53 to 62 Mt) of nickel supply simultaneously threatens the top 10% of priority areas for terrestrial and marine biodiversity conservation under the Announced Pledges Scenario.

Mining activities affect terrestrial, freshwater and marine biodiversity through numerous impact pathways and mechanisms, with aquatic pollution identified as a pervasive pressure^38^. Strip mining in tropical forests exposes bare ground and waste rock to erosion (Fig. 2), and heavy rainfall mobilizes metal-contaminated sediments that can travel tens to hundreds of kilometres downstream, persisting in freshwater systems for centuries or polluting nearshore marine environments^36,37,39^. While the absence of a globally consistent conservation priority layer precluded modelling of threats to freshwater biodiversity, recent assessments show that metal mining is degrading riverine ecosystems worldwide through increased sediment loads and long-distance contaminant transport derived from active and inactive mine sites^36^, in addition to artisanal and small-scale operations^40^. In Indonesia, current regulations do not require restoration to pre-mining conditions—emphasizing alternative post-mining land uses—and progressive rehabilitation remains rare, despite ongoing deforestation within mining concessions^41^. Without effective rehabilitation, cross-realm threats can persist after mining operations cease, particularly when sites are abandoned. Even with active restoration, forest degradation and biomass carbon loss can often only be partially reversed over reasonable timescales^42,43^. Given the potential growth of nickel mining in highly biodiverse coastal ecosystems in the tropics, subnational assessments of cross-realm threats are a critical research priority.

**Fig. 4:**
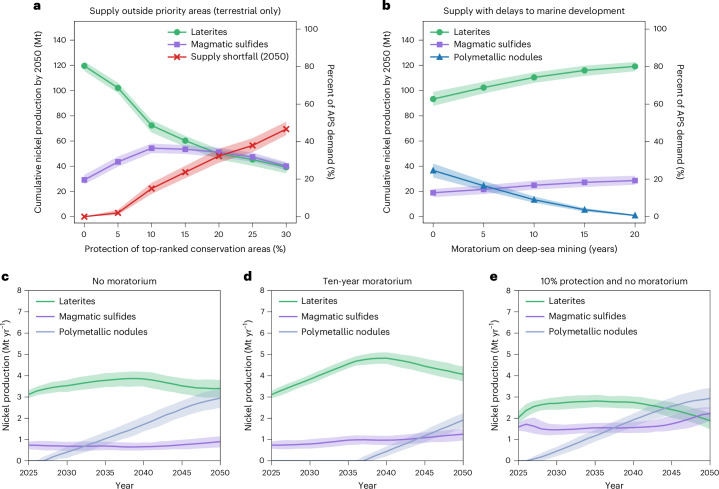
Influence of conservation actions on global nickel supply under the International Energy Agency’s Announced Pledges Scenario. **a**, Cumulative nickel production from laterite and magmatic sulfide deposits under scenarios of increasing protection of top-ranked terrestrial conservation areas from industrial mining and the potential for unmet demand (supply shortfalls) by 2050. **b**, Cumulative nickel production from terrestrial (laterites and magmatic sulfides) and marine (polymetallic nodules) deposits under scenarios of increasing delays to the potential start of deep-sea mining consistent with policies supporting a moratorium. **c**,**d**, We show annual nickel production under a rapid development scenario assuming no delay (**c**) versus a 10-year moratorium (**d**). **e**, We also model annual nickel production under a scenario of 10% protection with no moratorium on deep-sea mining, which avoids unmet demand. The line charts (**a**–**e**) show the median cumulative or annual nickel production across model simulations (*n* = 500), and shaded bands show the 5th to 95th percentile range.

Here we quantify the share of demand that could be met by only developing terrestrial deposits outside the most important global land areas for conserving biodiversity and storing carbon (Fig. 4a). These priority areas are where major new industrial activities should be carefully limited or avoided (for example, by implementing ‘no-go areas’ for mining) to achieve global conservation targets^17^. However, nickel deposits in these priority land areas contribute disproportionately to supply—even under a baseline scenario that assumes no growth in demand (Fig. 1e). Our modelling suggests that increasing protection of areas irreplaceable for biodiversity conservation and carbon storage substantially decreases production from laterite deposits and increases the risk of supply shortfalls before 2050 under the Announced Pledges Scenario (Fig. 4a). For example, avoiding mining in just the top 10% priority land areas leads to an ~47 Mt drop in nickel supply from laterites (from ~80% down to ~49% share of demand) and a shortfall of between 18 and 27 Mt (12 to 18% share of demand) by 2050 (Fig. 4a). However, this 10% protection scenario has substantial marine co-benefits, reducing supply by ~52 Mt from projects threatening the top 10% of marine conservation priorities (Fig. 4). Our modelling also suggests that magmatic sulfide deposits are often located outside the highest priority land areas and further inland from priority coastal ecosystems and could therefore respond positively to protections due to reduced competition with laterite deposits in the tropics (Fig. 4a and Extended Data Fig. 7d). Recent studies suggest demand-side signals could influence regional production priorities through responsible sourcing standards, policy shifts, downstream value chain transparency or a green premium to differentiate between ‘clean’ and ‘dirty’ nickel^7^. We highlight that prioritizing development outside of top-ranked conservation areas must be coupled with strong demand-side pressure, as supply-side measures alone (for example, ‘no-go areas’) could face considerable economic and geopolitical barriers by excluding major nickel producers. For example, the world’s largest nickel mine on Halmahera Island in Indonesia started production in 2020, yet the mine site lies within the top 1% of global priority areas for terrestrial biodiversity and stored carbon and is directly upstream of coastal waters ranking among the top 3–4% of global priority areas for marine biodiversity.

### The implications of deep-sea resource development

**Extended Data Fig. 8:**
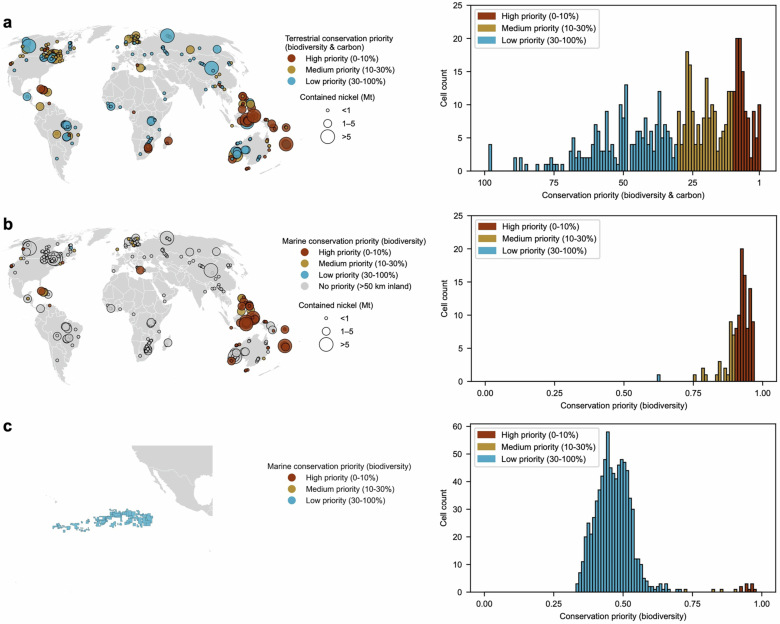
Spatial distribution of nickel deposits relative to terrestrial and marine conservation priorities. The intersecting cell value of each deposit on land was used to assign a terrestrial conservation priority (biodiversity and carbon) (**a**). The mean of intersecting cell values for deposits within 50 km of marine conservation priorities (biodiversity) was used to categorize threats to marine biodiversity priorities (**b**). The mean of intersecting cell values for exploration areas in the Clarion-Clipperton Zone was used to categorize threats to marine biodiversity priorities from polymetallic nodule resource development (**c**). Data for **a** from Jung et al.^17^, and data from **b** and **c** from Sala et al.^28^.

The Clarion-Clipperton Zone (CCZ) in the equatorial Pacific contains an estimated 274 Mt of nickel in polymetallic nodule deposits^19^—comparable to the sum of all identified land-based resources (Extended Data Fig. 7d). There are currently 17 active exploration areas for potential mining of polymetallic nodules in the CCZ located >400 km offshore from the nearest coastline (Extended Data Fig. 7a), with a larger area of seabed already under protection within 13 Areas of Particular Environmental Interest (Extended Data Fig. 7a)^23,44,45^. Our modelling suggests that polymetallic nodule deposits in the CCZ may contribute to long-term supply (Fig. 4c) if their high resource tonnage and grade are predictive of economic viability (which remains uncertain for commercial operations). Key exploration areas in the CCZ tend to occur further from priority areas for conserving marine biodiversity than several major laterite deposits on land (Extended Data Fig. 7a and Extended Data Fig. 8) based on current data^28^. Our modelling suggests polymetallic nodule mining could supply between 21 and 28% (31 to 42 Mt) of the total nickel required to meet demand under the Announced Pledges Scenario (Fig. 4b). Assuming this development pathway proceeds without delay, this represents the exploitation of less than 15% of the total estimated nickel contained within CCZ deposits. Furthermore, we find that the average nickel content of polymetallic nodules in the CCZ (~1.3%) is higher than the average mined ore grade on land (Extended Data Fig. 5).

The abyssal seafloor of the CCZ (~4–6 km depth) is a food-limited, low-biomass environment hosting more than 6,000–8,000 metazoan species—primarily small worms and crustaceans from the phyla Annelida, Nematoda and Arthropoda^23,46^. The lasting biodiversity impacts of deep-sea mining depend largely on the technology used. Modern systems disturb the top 3–5 cm of sediment, whereas long-term disturbance studies focus on systems that were more invasive, such as the Ocean Minerals Company (OMCO) mining test in 1979, which created 20–80 cm deep furrows^23,47–49^. The OMCO test caused long-lasting impacts on invertebrate communities living on the seafloor, while sediment-dwelling organisms and microbial communities had similar densities to undisturbed control sites^48^. Current evidence suggests impacts on nodule-dependent taxa are expected to be long-lasting across contract areas, and recovery will depend on whether there is sufficient hard substrate left unmined to support recolonization^23,48^. Two months following a recent industrial trial in 2022, sediment-dwelling macrofauna were approximately two-thirds of their baseline density and diversity in directly mined areas^47^. In nearby areas affected by sediment plumes, neither short-term nor long-term studies found significant decreases in faunal abundance compared to control sites^47,48^. We stress that any future polymetallic nodule mining requires effective and evidence-based environmental management to mitigate adverse effects on deep-sea biodiversity and ecosystem services^48,50^ and that achieving such outcomes may present a major challenge^51,52^. We also note that while the development of deep-sea projects may supplement a portion of supply from terrestrial sources, this does not guarantee a wholesale reduction in terrestrial impacts, as existing operators may still fail to scale back land-clearing efforts, manage downstream pollution and implement post-mine rehabilitation.

Our modelling suggests that delaying deep-sea resource development while increasing terrestrial production could have unintended consequences for biodiversity and decarbonization (Fig. 4). For example, we show a 10-year delay (Fig. 4c versus Fig. 4d) may lead to further nickel mining primarily in the tropics (and associated investments in processing capacity) to meet projected demand under the Announced Pledges Scenario (~17 Mt more from laterites, ~6 Mt more from magmatic sulfides, ~23 Mt less from polymetallic nodules; Fig. 4b). Our modelling highlights that it is possible to fill the supply shortfall created by protecting the top 10% of terrestrial conservation priority areas under a scenario including polymetallic nodule resource development (Fig. 4e). We emphasize that a deep-sea mining moratorium offers no guarantee that higher-cost, potentially more ecologically responsible land-based producers will secure sizeable market share. Even if these higher-cost land-based producers gain traction in the near-term, polymetallic nodule mining could later exert market pressures similar to those currently seen from low-cost Indonesian laterites should it ultimately prove cost-effective^53^. In a potential worst-case outcome, efforts are made to fast-track the development of many smaller lower-grade terrestrial deposits in a rush to secure nickel supply, only for these mines to become stranded assets and environmental liabilities if they cannot compete.

### Securing ecologically responsible nickel

Ecologically responsible mining requires protecting regions of global importance for biodiversity and climate targets^54^, such as avoiding large-scale mine development or expansion in intact tropical forest ecosystems^42^. Our modelling highlights that, by 2050, nickel mining could lead to extensive ecological erosion and biomass carbon loss in some of Earth’s most biodiverse regions, increasing the risk of plant and animal extinctions. Establishing ‘no-go areas’^55^ in regions identified as having the highest conservation importance—especially those with less favourable economic prospects or poor reporting standards—could lead to positive outcomes for both decarbonization and conservation goals. That said, avoiding mine development in nickel-rich regions will be particularly challenging due to complex geopolitics associated with national interests in securing critical mineral supply, especially without a viable alternative resource stream. Furthermore, nickel mining offers resource-rich developing countries a vital source of income and employment, although overreliance can leave local communities vulnerable to market disruptions. Ensuring the adoption of standards for ecologically responsible mining and sustainability reporting could improve measures to mitigate mining impacts^54^. However, currently half of global mining areas lack basic production information necessary for independent impact assessments. This is particularly prevalent in Russia, China, Indonesia and Brazil^56^.

**Extended Data Fig. 9:**
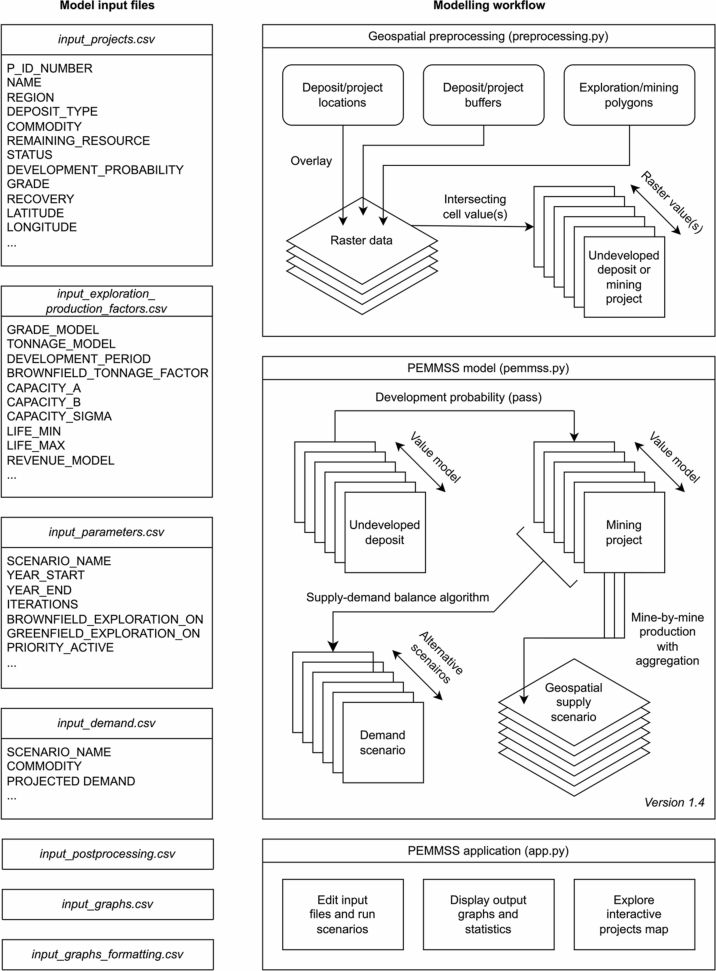
The PEMMSS model input files, workflow and web application. The input files are provided in the Supplementary Data to reproduce the results. The PEMMSS model is implemented in Python^25^, and Version 1.4 includes additional features for handling geospatial data and an application developed using the Shiny package to display results^59^. The results of the geospatial preprocessing are presented in Extended Data Fig. 8.

Current pathways to decarbonization require a substantial increase in nickel supply over the coming decades, although the pace of demand growth will depend on policy ambitions and technological developments. Our scenario modelling (Extended Data Fig. 9) highlights that both demand-side responses and industry-wide initiatives are needed to mitigate the impacts of mining in globally important areas for conserving biodiversity and storing carbon. Companies operating in high-priority conservation areas must maintain transparency and accountability across their value chain to ensure mined nickel supports only the most critical end uses while enabling manufacturers to make informed sourcing decisions. Ecologically responsible mining also requires extending the mitigation hierarchy to manage offsite and indirect impacts^54^, especially given our finding that over half of nickel production by 2050 may come from coastal mines upstream of global strongholds of marine biodiversity (Fig. 3). Targeted investments for mitigating non-mining threats may help offset unavoidable impacts in high-priority conservation areas^57^, but these initiatives are rare and require robust safeguards and independent validation to ensure intended outcomes^58^. Increasing exploration and investment in ecologically responsible terrestrial projects and accelerated research and development into the economic viability and environmental risks of deep-sea mining is clearly critical for minimizing threats to global conservation priorities over the next decade. Our results show that increasing terrestrial production while delaying the potential development of deep-sea resources is likely to lead to further mine expansion in the tropics within areas that are irreplaceable for conservation. Our goal here is not to advocate for one form of future development over another but rather to highlight the enormous stakes for biodiversity contingent on land and deep-sea conservation policy and our ability to understand the forces influencing global mine development. Future supply decisions must be grounded in integrated research that balances sustainability trade-offs and biodiversity risks across terrestrial, coastal and deep-sea ecosystems.

## Methods

### Supply scenario model

The Primary Exploration, Mining and Metal Supply Scenario (PEMMSS) model was used to develop the scenarios presented in this Article (Extended Data Fig. 9). The PEMMSS model provides a way to translate scenarios for mineral commodity demand into a more nuanced and stochastic understanding of how this supply could be met through mine development and exploration^25^. The model tracks cohorts of individual mines and deposits through time, incorporating uncertainties associated with mine development, achievable production rates, deposit characteristics and relative deposit value. Each model run produces different results due to these uncertainties. The path-dependency associated with the specific collection of mines developed in each model run and the supply outcomes over time are preserved in model outputs, enabling detailed interrogation of modelled scenarios. As part of this, most model parameters can be specified for individual mining projects and mineral deposits that are seeded into the model. More generalized assumptions for model parameters can also be specified for individual regions and deposit types. Although not used as part of the present study, the modelling framework also has functionality to stochastically model mineral deposit discovery and exploration outcomes, using grade-tonnage distributions for deposit types and uncertainty factors for resource discovery across regions (which can be linked to undiscovered deposit density or resource tonnage estimates). For further details on the modelling framework, we refer readers to the open-access publication that describes the full methodology, design rationale and calculation framework underpinning the PEMMSS model^25^.

An open-source, Python-based implementation of the PEMMSS model is available on GitHub and Zenodo^59^. As part of this study, we extended the model codebase to allow more explicit specification and handling of spatial data through the addition of coordinates for individual mines and deposits, optimization of spatial routines, and the ability to work with geopackages. These extensions were incorporated into a new release of the model, PEMMSS version 1.4, which also includes some memory and runtime optimizations and ease-of-use improvements such as a graphical user interface developed using the Python Shiny package^59^. The initial conditions, parameters and data used to define scenarios in the PEMMSS model are specified using a collection of comma-separated value (CSV) files described below. The scenarios presented in this study are defined in Extended Data Table 1, each with its own folder identifier with all the input files required to reproduce the results (Supplementary Data).

### Model parameters and data

The various primary nickel demand scenarios shown in Extended Data Fig. 1 were passed to the model using the input_demand.csv file (Extended Data Fig. 9). We modelled supply under the International Energy Agency’s Global Critical Minerals Outlook 2025 scenarios^3^ using linear interpolation between available data points (Extended Data Fig. 1). The International Energy Agency’s nickel demand projections vary substantially across three scenarios: (1) the Stated Policies Scenario (STEPS), which reflects the current trajectory based on existing policies; (2) the Announced Pledges Scenario (APS), which assumes governments fully meet their announced climate targets, including net zero pledges and (3) the Net Zero Emissions by 2050 Scenario (NZE), which provides a pathway to net zero emissions by 2050. To provide an upper bound on demand projections, we modelled the capacity of primary (mined) supply to meet the International Energy Agency’s total demand projections. The contribution of secondary supply to meeting projected total demand is not made available across all International Energy Agency scenarios but is expected to make up less than a 3% share by 2030 and less than 10% by 2040 under the Announced Pledges Scenario^3^. To provide a conservative lower bound on demand projections, we included an additional Baseline Reference Scenario (REF), which assumes no further growth in nickel demand beyond the estimated nickel production in 2024 of 3.7 Mt (Extended Data Fig. 1).

Data for individual nickel deposits and mines were passed to the model using the input_projects.csv file (Extended Data Fig. 9). This includes estimates of the resource tonnage, ore grade, deposit type and initial status from the S&P Capital IQ Pro Database for known nickel deposits as shown in Extended Data Fig. 2a. The S&P Database may contain incomplete data, particularly relating to production, due to issues of transparency affecting large portions of the mining industry. This may result in an underrepresentation of total localities affected by nickel supply, particularly those that contain informal mining operations^56^. Nonetheless, this database is considered the most viable for global-scale metals and mining studies^10,13^, and we have primarily sourced resource tonnage and grade information from this database, which has better representation. Furthermore, we have validated resource tonnage and grade data with the most recent global study of reported nickel deposits and projects (Extended Data Fig. 2b).

To meet a projected supply gap in any given year, the PEMMSS model selects potential projects prioritized by their relative value^25^. We classified global terrestrial deposits as either laterites or magmatic sulfides (including hydrothermal deposits), consistent with recent studies^13^, noting that other deposit types exist but do not contribute materially to terrestrial resource estimates^26^. The likelihood that a selected project will commence production in a given year if there is a supply shortfall is then determined by its development stage, with assigned probabilities of 10% for early exploration and feasibility and 25% for late exploration and construction. The initial status and development probability for each project was assigned based on the reported activity status and development stage in the S&P Capital IQ Pro Database. The development probabilities allowed the ensemble of simulations to explore a greater number of potential path dependencies across iterations^25^ and simulate realistic lead times for projects in early versus late development stages^60^.

Regression coefficients that relate mineral reserve size to mine production capacity were passed to the model using the input_exploration_production_factors.csv file (Extended Data Fig. 9). We used Taylor’s rule—widely used for scoping and feasibility studies in the mining industry—to estimate ore production capacity (*P*):

$$P=a\times {T}^{b}$$

where *T* is the ore tonnage (that is, identified or remaining resource including reserves in Extended Data Fig. 2a), and *a* and *b* are coefficients to be estimated^25,61,62^. We used the generalized coefficients derived from a dataset of open-pit and underground (block cave) mines for a range of different primary metal commodities and deposit types^61^ to simulate annual production capacity and mine life, assuming 350 operating days per year (ref. ^62^). Production capacity is also constrained by a minimum and maximum mine life of 5 and 50 years, respectively (Extended Data Fig. 3). From this, the PEMMSS model estimates mine-by-mine commodity supply (*S*) given by

$$S=P\times G\times R$$

where *G* is the average reported ore grade of a deposit (Extended Data Fig. 2a) and *R* is the estimated recovery rate after processing. We estimated recovery rates (*R*) for each deposit using a relationship derived from a comprehensive nickel resource and production dataset, which includes variation by deposit type and ore grade^26^. The mine-by-mine commodity supply (*S*) was used to estimate the relative value of each deposit and the aggregate production across regions and deposit types (Fig. 1). All reported results for mine production, grade and status include uncertainty bounds, including the 5th to 95th percentiles as range estimates to minimize the influence of outliers (that is, 90% of simulations fall within this range).

We conducted a series of geospatial analyses to determine the spatial overlap between mining projects and priority conservation areas and estimate the quantity of nickel produced from within or near these areas (Extended Data Fig. 8 and Extended Data Fig. 9). First, we sampled the point locations of mines and deposits^13^ to extract terrestrial conservation priority rank values from a global 10-km resolution map of global land areas ranked by their importance for conserving biodiversity and storing carbon^17^. A buffer radius of between 1 and 10 km has been used in recent studies to estimate a nickel mine’s direct environmental impacts (not including indirect impacts)^13^. Therefore, we classified each mine or deposit by whether its potential direct footprint occurs within high (top 10%), medium (10–30%) and low (>30%) priority areas, which were then passed to the PEMMSS model through the input_projects.csv file (Extended Data Fig. 9). Next, we classified mines into two groups: coastal mines (where point locations are within 50 km of a coastline) and inland mines. A buffer radius of 50 km has been used to capture the potential influence of both direct and indirect impacts of mining on biodiversity^10^. Therefore, for coastal mines, we generated a 50 km buffer and calculated the mean priority value of all cells touching the buffer from a 50 km resolution global map of marine biodiversity conservation priorities^28^. We then classified coastal mines as being within 50 km of high-, medium- or low-priority areas for conserving marine biodiversity. We note that although both terrestrial and marine priority maps have coarse resolution, they use the best available data on species distribution, with particular attention to biases introduced by incomplete knowledge on distribution of species in selected global regions (the so-called Wallacean shortfall) and are therefore considered fit-for-purpose for global-scale analyses^17,28^. Nevertheless, subnational studies would require finer-scale species and carbon data. All geospatial analyses were performed in Python using the geopandas, rasterio and rasterstats packages. The terrestrial and marine conservation priority areas are shown in Fig. 1d and Extended Data Fig. 7a, respectively.

### Model sensitivity analyses

In our study, we primarily consider the International Energy Agency’s Announced Pledges Scenario, providing well-recognized demand estimates, reflecting announced targets and ambitions for decarbonization^3^. However, we tested the sensitivity of our supply scenarios to alternative demand scenarios (Extended Data Fig. 1), as shown in Fig. 1c,e and Extended Data Fig. 4. We also tested the sensitivity of our results to alternative value models, including the contained nickel in a deposit (*C* = *T* × *G*), a deposit’s ore grade alone (*G*) and ore tonnage alone (*T*). We included three further sensitivity analyses where: (1) existing projects are prioritized ahead of undeveloped deposits to identify an upper bound of supply from existing mines versus undeveloped deposits; (2) remaining ore tonnage for active projects expands by 5% per year to simulate increasing brownfield exploration and (3) each deposit is stochastically assigned a random relative value to provide a benchmark for comparison.

Our sensitivity analysis of future supply to alternative value models indicates that deposit characteristics (ore tonnage and grade) are a major driver of recent shifts towards mining of laterite ore bodies in the tropics (Extended Data Figs. 2 and 4). The recent surge in low-cost supply from Indonesia suggests that deposit characteristics, coupled with other economic drivers, have allowed it to capture substantial market share (Extended Data Figs. 2 and 6). These conditions have led to competition with higher-cost projects in other regions. Recent low nickel prices mean that countries with higher production costs are also less likely to invest in brownfield and greenfield exploration^3^.

Deep-sea resources are included in our study to demonstrate the vast scale of any unconventional resource stream—terrestrial or marine—that would be necessary to influence current trends of nickel mine expansion in the tropics over the coming decades. We limited our analysis to the implications of rapid versus delayed polymetallic nodule mining (Fig. 4b) to understand potential changes in global nickel supply dynamics and the likely spatio-temporal distribution of mining projects on land. Our analysis of the sensitivity of nickel supply to deep-sea mining delays (Fig. 4b) used the PEMMSS model’s background discoveries feature^25^. This feature adds one new marine deposit per year to our resource dataset where each project is stochastically assigned 100 to 400 million dry tonnes of polymetallic nodules at an average nickel grade of between 1.2 and 1.5% (refs. ^19,63^). The average nickel content of polymetallic nodules varies by ocean region^19^, and, as such, our model only considers marine resource development within designated exploration areas in the CCZ of the equatorial Pacific Ocean (as shown in Extended Data Fig. 7a). Recent studies have estimated total contained nickel in polymetallic nodule deposits in the CCZ at 274 Mt, with global estimates potentially far greater at an estimated 1.25 Gt in polymetallic nodules and 3.23 Gt in ferromanganese crusts^64^. We used the same production capacity relationship as for laterites and magmatic sulfides to estimate annual production for each deep-sea mining operation (Extended Data Fig. 3). Although estimates of production capacity for nodule mining are uncertain, our simulations result in an average mine life of 20 years for polymetallic nodule deposits (with each operation ranging between 11 and 39 years depending on the assigned ore tonnage and grade), consistent with current pre-feasibility studies for commercial-scale operations^65,66^ and the term of an exploitation contract with the International Seabed Authority^67^. After the first marine deposit is assigned, each project will only start if it has a higher relative value than all other terrestrial deposits in that same year. We applied an initial development period of 2 years and a development probability of 50% for marine deposits, which leads to an estimated start date for deep-sea mining of between 2027 and 2030 (assuming no moratorium on deep-sea mining). Then, we incrementally applied 5-year delays to the initial development period to test the sensitivity of global supply to increasing lead times that may arise from policies supporting a moratorium (Fig. 4b).

### Model limitations

There are some limitations of the PEMMSS modelling framework that should be considered when interpreting the results of this study. For instance, accurately modelling long-term ore grade dynamics and outcomes is exceptionally difficult. The PEMMSS model only considers the average grade of identified mineral resources for each deposit. More complex economic feedbacks related to changing commodity prices influencing the economic cut-off grades used for deposit resource definition and mining are not incorporated. The PEMMSS model can approximate this type of information in a simple way through the ‘ore tranche’ functionality that is already implemented in the modelling framework, which allows specification of multiple tranches of available ore for each deposit that have separate assumed grades and cost/value functions. However, existing datasets available for nickel deposits do not have the level of detail required to make full use of this model functionality. Due to this, Extended Data Fig. 5, showing the amount of ore mined, the average mined ore grade and the number of new mines starting, may understate the full range of potential long-term outcomes for these model outputs.

Another limitation is the bias that may be embedded in the regression coefficients used to relate mineral resources to production capacity. These were derived from a dataset containing 539 mines with primary commodities including gold, copper, lead-zinc and nickel^61^. Due to some similarities in deposit geomorphology and mining methods, these parameters may be reasonable proxies for nickel sulfide deposits. However, data for these relationships are less developed for nickel laterite deposits that have different geomorphology and production constraints. Resource reporting quality varies by jurisdiction^56^ and established mining regions may have well-documented resources that are unlikely to be developed due to factors beyond the scope of our model (for example, permitting constraints, community opposition or insufficient processing capacity), potentially leading to overestimates of production. Conversely, emerging frontiers such as Indonesia may have limited or delayed reporting that does not capture ongoing exploration, potentially leading to underestimates of production. Likewise, there are related data limitations and uncertainties in operational mining methods that inhibit estimations of potential production capacity from seafloor deposits. Due to this, the estimated production capacity across the set of known, undeveloped nickel deposits is particularly uncertain and so results should be interpreted carefully on this basis. To provide a reasonable constraint on production capacity estimates, we have implemented a minimum and maximum mine life of 5 and 50 years, respectively (Extended Data Fig. 3)^25^.

Our analysis focused exclusively on nickel as the primary commodity and did not consider potential co-products (such as copper and cobalt) present in both terrestrial and deep-sea resources^19,26^. Reprocessing of tailings deposits of former projects was not considered in our study. These deposits represent an estimated 0.49 Mt of nickel^26^ and hence their inclusion would not materially affect our modelling results. Additionally, while we include known nickel resources in the Clarion-Clipperton Zone^19^, we did not consider polymetallic nodule deposits outside this region or other types of deep-sea deposits such as seafloor massive sulfides or cobalt-rich ferromanganese crusts^64^, which could also contribute to future nickel supply but are in earlier stages of development.

The terrestrial conservation priority maps used in our analysis represent the most comprehensive global integration of biodiversity and carbon data to date, including all extant terrestrial vertebrates (5,685 mammals, 6,660 amphibians, 10,953 birds and 10,585 reptiles), a representative subset of all accepted vascular plant species (~41%, 193,954 species) and the best available maps of aboveground and belowground biomass carbon and vulnerable soil carbon^17^. However, the study notably lacks freshwater, soil and invertebrate species for which data are currently insufficient for global-scale analyses. Similarly, the ocean priority maps represent the most comprehensive global assessment of priorities for conserving marine biodiversity, integrating multiple data sources^28^. Whereas each study employs realm-specific methodologies for its global optimization, both consider species threat status, integrate existing protected areas and conduct extensive uncertainty analyses^17,28^. Although any one analysis has its limitations, multiple global-scale studies confirm that the land and coastal ecosystems of top nickel producers—including Indonesia (Wallacea), the Philippines and New Caledonia—are global hotspots of terrestrial and marine species endemism^68–70^. We consider the maps used in our analysis appropriate for global-scale analyses of mining threats, but emphasize that focused subnational assessments would be required to evaluate impacts on individual species and ecosystems.

While considerable progress has been made in recent years to map the direct footprint of global mining^56^, substantial uncertainty remains regarding indirect and offsite impacts. Available evidence suggests mining poses threats to ecosystems well beyond site boundaries^38,41^. For example, Sonter et al.^10^ show that mining-induced deforestation in the Brazilian Amazon can extend up to 70 km from lease boundaries, and Macklin et al.^36^ highlight that sediment-associated contaminants can travel 10–100 km downstream. Nickel laterite operations are often expansive and require extensive coastal infrastructure that directly affects local marine environments, exemplified by the construction of major coastal industrial parks in Indonesia in the past decade^15^. In our study, we applied a 50 km buffer to identify deposits with the potential to threaten marine conservation priorities (Fig. 3 and Extended Data Figs. 7 and 8). This threshold is constrained by the ~50 km resolution of available marine priority data^28^, ensuring at least one raster cell is sampled but is nonetheless consistent with buffers used in recent global mining threat assessments^10^. Whereas global mine-by-mine depletion is explicitly represented by our supply scenarios, we do not model the persistence or reduction of threats to conservation priorities following mine closure. Ultimately, global prioritization studies such as ours should be used to identify areas where regional and local impact assessments may be particularly effective for informing conservation action.

While we have modelled how deposit characteristics and conservation priorities may influence mine development, we cannot predict what a diverse array of mine actors will choose to do in response to changing demand signals, and so the question of whether marine resources would substitute for, partially replace or simply add to terrestrial supply remains unresolved. Fully understanding these dynamics—and counterfactual scenarios of continued fossil fuel production and climate change—is critical to provide sound recommendations for delivering optimal biodiversity outcomes^11^.

### Reporting summary

Further information on research design is available in the Nature Portfolio Reporting Summary linked to this article.

## Supplementary information

Reporting Summary

Peer Review File

Supplementary Data Input files required to reproduce the PEMMSS model results. The input files and model workflow are summarized in Extended Data Fig. 9.

## Figures and Tables

**Extended Data Table 1:**
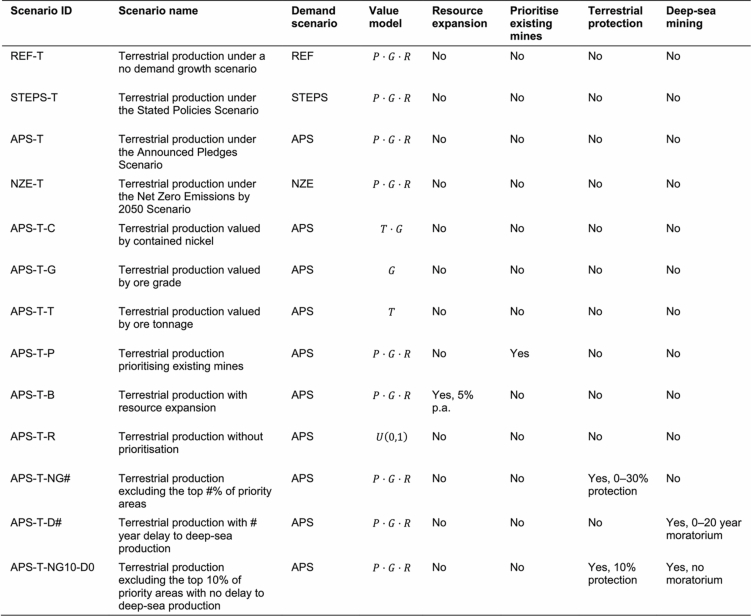
Input parameters for scenarios and sensitivity analyses

Input parameters for scenarios and sensitivity analyses.

## Data Availability

The dataset and input files required to reproduce the results of the PEMMSS model are available in the Supplementary Information.

## Code availability

All code required to run the PEMMSS model (version 1.4) is available via Zenodo at 10.5281/zenodo.16792366 (ref. ^59^).
