# Supplementary material for: Growing nickel supply from the tropics threatens priority conservation areas
Source: Nat Ecol Evol. 2026 May 6;10(7):1305–15. doi: 10.1038/s41559-026-03068-4 (PMC13345959; doi:10.1038/s41559-026-03068-4)
Supplement: Supplementary file 1 — Reporting Summary [file 41559_2026_3068_MOESM1_ESM.pdf]

nature portfolio

Corresponding author(s): Jayden Hyman

Last updated by author(s): 18/03/2026

Reporting Summary

Nature Portfolio wishes to improve the reproducibility of the work that we publish. This form provides structure for consistency and transparency in reporting. For further information on Nature Portfolio policies, see our Editorial Policies and the Editorial Policy Checklist.

Statistics

For all statistical analyses, confirm that the following items are present in the figure legend, table legend, main text, or Methods section.

n/a	Confirmed	
☐	☒	The exact sample size (*n*) for each experimental group/condition, given as a discrete number and unit of measurement
☒	☐	A statement on whether measurements were taken from distinct samples or whether the same sample was measured repeatedly
☒	☐	The statistical test(s) used AND whether they are one- or two-sided<br>*Only common tests should be described solely by name; describe more complex techniques in the Methods section.*
☒	☐	A description of all covariates tested
☒	☐	A description of any assumptions or corrections, such as tests of normality and adjustment for multiple comparisons
☐	☒	A full description of the statistical parameters including central tendency (e.g. means) or other basic estimates (e.g. regression coefficient) AND variation (e.g. standard deviation) or associated estimates of uncertainty (e.g. confidence intervals)
☒	☐	For null hypothesis testing, the test statistic (e.g. *F*, *t*, *r*) with confidence intervals, effect sizes, degrees of freedom and *P* value noted<br>*Give P values as exact values whenever suitable.*
☒	☐	For Bayesian analysis, information on the choice of priors and Markov chain Monte Carlo settings
☒	☐	For hierarchical and complex designs, identification of the appropriate level for tests and full reporting of outcomes
☒	☐	Estimates of effect sizes (e.g. Cohen's *d*, Pearson's *r*), indicating how they were calculated

Our web collection on statistics for biologists contains articles on many of the points above.

Software and code

Policy information about availability of computer code

Data collection	Resource data was obtained from the S&P Capital Pro IQ Database and scientific literature cited in the paper.
Data analysis	An open-source implementation of the Primary Exploration, Mining and Metal Supply Scenario (PEMMSS) model (version 1.4) was used for model analyses. The PEMMSS model code is available on GitHub (https://github.com/sanorthey/pemmss) and Zenodo (https://zenodo.org/records/16792366). Geospatial analyses were performed in Python (version 3.12) using geopandas (version 1.1.2), rasterio (version 1.5.0), and rasterstats (version 0.20.0).

For manuscripts utilizing custom algorithms or software that are central to the research but not yet described in published literature, software must be made available to editors and reviewers. We strongly encourage code deposition in a community repository (e.g. GitHub). See the Nature Portfolio guidelines for submitting code & software for further information.

Data

Policy information about availability of data

All manuscripts must include a data availability statement. This statement should provide the following information, where applicable:

- Accession codes, unique identifiers, or web links for publicly available datasets
- A description of any restrictions on data availability
- For clinical datasets or third party data, please ensure that the statement adheres to our policy

We used a global dataset of 487 nickel deposits compiled from the S&P Capital IQ Pro Database and recent literature on global nickel resources. The dataset

includes information on resource tonnage, grade, deposit type, development stage and location. The input files required to reproduce the results of the PEMMSS model are available in the supplementary information.

Research involving human participants, their data, or biological material

Policy information about studies with human participants or human data. See also policy information about sex, gender (identity/presentation), and sexual orientation and race, ethnicity and racism.

Reporting on sex and gender	N/A
Reporting on race, ethnicity, or other socially relevant groupings	N/A
Population characteristics	N/A
Recruitment	N/A
Ethics oversight	N/A

Note that full information on the approval of the study protocol must also be provided in the manuscript.

Field-specific reporting

Please select the one below that is the best fit for your research. If you are not sure, read the appropriate sections before making your selection.

☐ Life sciences ☐ Behavioural & social sciences ☒ Ecological, evolutionary & environmental sciences

For a reference copy of the document with all sections, see nature.com/documents/nr-reporting-summary-flat.pdf

Ecological, evolutionary & environmental sciences study design

All studies must disclose on these points even when the disclosure is negative.

Study description	We used a global mine-by-mine supply scenario model (PEMMSS v1.4) to investigate how projected nickel demand (2025–2050) could be met from known terrestrial and deep-sea resources. Our study quantifies trade-offs between securing sufficient nickel supply for decarbonisation and protecting priority conservation areas. The maps of terrestrial and marine priority conservation areas were derived from existing literature and associated public repositories, including Jung et al. 2021 (https://doi.org/10.5281/zenodo.5006332) and Sala et al. 2021 (https://doi.org/10.25349/D9N89M), respectively.
Research sample	A global dataset of 487 nickel deposits and reported resources.
Sampling strategy	N/A
Data collection	No new data were collected.
Timing and spatial scale	Nickel supply was modelled between 2025 and 2050. Deposit information was collated based on the most recent reported data up to the year 2025, with global spatial coverage. The terrestrial and marine priority conservation area maps have a resolution of 10 km and 50 km, respectively.
Data exclusions	Deposits were excluded if they lacked essential resource information required by the PEMMSS model.
Reproducibility	The code required to run the PEMMSS model (version 1.4) is available at the public Zenodo repository: https://doi.org/10.5281/zenodo.16792366
Randomization	N/A
Blinding	N/A

Did the study involve field work? ☐ Yes ☒ No

Reporting for specific materials, systems and methods

We require information from authors about some types of materials, experimental systems and methods used in many studies. Here, indicate whether each material, system or method listed is relevant to your study. If you are not sure if a list item applies to your research, read the appropriate section before selecting a response.

Materials & experimental systems

n/a	Involved in the study
☒	☐ Antibodies
☒	☐ Eukaryotic cell lines
☒	☐ Palaeontology and archaeology
☒	☐ Animals and other organisms
☒	☐ Clinical data
☒	☐ Dual use research of concern
☒	☐ Plants

Methods

n/a	Involved in the study
☒	☐ ChIP-seq
☒	☐ Flow cytometry
☒	☐ MRI-based neuroimaging

Plants

Seed stocks: N/A

Novel plant genotypes: N/A

Authentication: N/A
