# Supplementary material for: Growing nickel supply from the tropics threatens priority conservation areas
Source: Nat Ecol Evol. 2026 May 6;10(7):1305–15. doi: 10.1038/s41559-026-03068-4 (PMC13345959; doi:10.1038/s41559-026-03068-4)
Supplement: Supplementary file 2 — Peer Review File [file 41559_2026_3068_MOESM2_ESM.pdf]

nature portfolio

Peer Review File

Growing nickel supply from the tropics threatens priority conservation areas

Corresponding Author: Dr Jayden Hyman

Version 0:

Decision Letter:

23rd December 2025

*Please ensure you delete the link to your author homepage in this e-mail if you wish to forward it to your co-authors.

Dear Jayden,

[redacted]

Your manuscript entitled "Growing nickel supply from the tropics threatens priority conservation areas" has now been seen by four reviewers, whose comments are attached. The reviewers have raised a number of concerns which will need to be addressed before we can offer publication in Nature Ecology & Evolution. We will therefore need to see your responses to the criticisms raised and to some editorial concerns, along with a revised manuscript, before we can reach a final decision regarding publication.

We therefore invite you to revise your manuscript taking into account all reviewer and editor comments. Please highlight all changes in the manuscript text file.

We are committed to providing a fair and constructive peer-review process. Do not hesitate to contact us if there are specific requests from the reviewers that you believe are technically impossible or unlikely to yield a meaningful outcome.

When revising your manuscript:

* Include a “Response to reviewers” document detailing, point-by-point, how you addressed each reviewer comment. If no action was taken to address a point, you must provide a compelling argument. This response will be sent back to the reviewers along with the revised manuscript.

* If you have not done so already please begin to revise your manuscript so that it conforms to our Article format instructions at http://www.nature.com/natecolevol/info/final-submission. Refer also to any guidelines provided in this letter.

* Include a revised version of any required reporting checklist. It will be available to referees (and, potentially, statisticians) to aid in their evaluation if the manuscript goes back for peer review. A revised checklist is essential for re-review of the paper.

* Extended Data Figures - please ensure that any supplementary figures and tables that are crucial to the manuscript’s conclusions are converted into Extended Data figures and tables to increase visibility of these data. Extended Data figures and tables are online-only (present in the online PDF and full-text HTML versions of the paper), peer-reviewed display items that provide essential background to the article but are not included in the main article due to space constraints. A maximum of ten Extended Data display items (figures and tables) is permitted.

Please use the link below to submit your revised manuscript and related files:

Link Redacted

Note: This URL links to your confidential home page and associated information about manuscripts you may have submitted, or that you are reviewing for us. If you wish to forward this email to co-authors, please delete the link to your homepage.

We hope to receive your revised manuscript within four to eight weeks. If you cannot send it within this time, please let us know. We will be happy to consider your revision so long as nothing similar has been accepted for publication at Nature Ecology & Evolution or published elsewhere.

Please do not hesitate to contact me if you have any questions or would like to discuss these revisions further.

We look forward to seeing the revised manuscript and thank you for the opportunity to review your work.

[redacted]

Reviewer expertise:

Reviewer #1: environmental conflicts in minerals

Reviewer #2: mining impacts

Reviewer #3: integrated assessments, biodiversity impact assessments, spatial analysis, mining impacts

Reviewer #4: critical minerals, material flow analysis

Reviewers' comments:

Reviewer #1 (Remarks to the Author):

This is an excellent article that uses specific demand scenarios for a critical metal for the green transition and links that to key economically viable reserves in the tropics. Such an approach can assist in considering broader prioritization of supply sources be subsequently using tools such as Life Cycle Analysis.

The rise in supply from lateritic sources rather than magmatic sulfides has been under-studied. There are three areas where revisions would be needed before publication:

a) The rise in supply scenarios for magmatic sulfides closer to 2050 in figure 1b needs to be better explained. There are major exploration projects underway and also new techniques to harness lower grade sulfide ores. The authors should consult with the International Nickel Study Group in Portugal and the Nickel Institute in this regard to test the validity of their projections.

b) The authors have too casually dismissed the secondary source projections based on an assumption of current supply being locked in infrastructure. However, nickel has been used in steel production for over a century and there are indeed many supply sources which hitherto have perhaps been neglected. For example, the "ghost cities of China" and other sources of nickel in ageing infrastructure from urban mining needs to be acknowledged. Refer to the following study https://www.nature.com/articles/s41467-020-15246-4 as a starting point.

c)The authors should also acknowledge that some of the arguments for mining pertain to the economic development impact on the extractive countries. In particular Indonesia and the Philippines which see this as a major source of income for national accounts. Some discussion of alternative development paths for these countries should also be noted (they clearly have potential for various service sector economies as well).

With these changes this article would be ready for publication

Reviewer #2 (Remarks to the Author):

This is a very well-written, comprehensive and topical manuscript outlining the potential impacts of nickel mining on priority conservation areas. The modelling conducted considers a wide variety of issues related to nickel sulphide, laterite and deep sea nodule mining and presents scenarios for these related to biodiversity for the coming decades. Overall, the manuscript is excellent and I recommend acceptance following very minor revision and consideration of the following comments:

Line 114. - 'an estimated 55 to 96 new nickel mines starting produciton by 2050'. How close to reality are these estimates, given the length of time it can take (10-20 years) to get a mine up and running?

Line 115 - Please define what you mean by 'secondary supply'. If this does not include tailings recycling then please add the estimate of Ni recovery from this secondary supply to your estimates in these sentences. If the estimates for Ni recovery from tailings is large, please state how this would affect your modelling and conclusions.

Line 182. Please change 'tailings dams' to 'tailings storage facility' (and then change the 'be stored in' to something like 'be deposited in'. Also - what is your reference for the 'billions of tones of waste'? Has this number been properly modelled?

Line 184. Please change' tailings dam failures' to 'tailings storage facility failures'. Also change 'pollution' to 'contamination' (pollution is contamination that has a demonstrated negative effect on human health).

Reviewer #2 (Remarks on code availability):

I am not familiar enough with modelling to run and understand codes like this.

Reviewer #3 (Remarks to the Author):

Hyman et al. present a prospective analysis of potential conflicts between Nickel mining and priority conservation areas, including both terrestrial and deep-sea mining. I greatly enjoyed the paper, which is very clearly written and among the first analyses to compare potential impacts on biodiversity from terrestrial and deep-sea sources, even if in a simplified overlay. I have identified below a number of issues that I hope will help strengthen the paper before acceptance.

Main comments

After a mine closes, impacts can vary significantly depending on the country and type of mine. Moreover, once a deposit is fully exploited, the lasting impacts on biodiversity will differ greatly between terrestrial and marine mines. I suggest the authors expand the paragraph starting at L141 to include a more in-depth discussion of their findings in the context of post-mining impacts across different ecosystems.

While I appreciated the detailed discussion on the limitations of the PEMMSS approach, I missed context regarding the limitations of the biodiversity data from Jung et al. and how these limitations influence the results presented in this manuscript. I encourage the authors to discuss potential biases and the representativeness of the dataset used to identify priority conservation areas. Additionally, it would be helpful to clarify how these factors relate to existing protected areas and other commonly used metrics, such as threat status, and to what extent freshwater ecosystems are represented by these layers.

Many of the marine impacts rely on the assumption that mines within 50 km of coastlines contribute to marine impacts. First, for land mines, the radius used to identify overlap with conservation priority areas is 1 to 10 km, which is inconsistent with the 50 km radius. Second, what is the rationale behind the impact mechanisms for land or coastal mines affecting marine biodiversity? It is unclear how the link to the 50 km radius of indirect impacts from Sonter et al. (which remains, in any case, very arbitrary and unverified) indicates how a mine connects land to the coast. I encourage the authors to better support this assumption by explaining the impact pathways that justify using a 50 km radius for land-to-marine impacts. If pollution is the main concern, they could consider using drainage lines with a justified distance threshold (since not all mines within 50 km of the coast might connect to it, depending on local terrain). If the authors decide to maintain a buffer approach, I recommend at least conducting a sensitivity analysis using 10 km and 50 km, as in Sonter et al. (2020).

Minor comments

L158: Depending on the screen, the 100% shade is barely visible. Please increase the contrast or use contours. The same applies to Figure 2.

L152-163: It is unclear what the “-T" represents in the scenario acronyms just by looking at the figure and caption. I need to get to the Supplement to learn that it refers to terrestrial. Please spell it out in the caption.

L141-143: How did the authors handle mine closure in their impact assessment modeling? If a mine closes, does the impact continue? In that case, is the mine still included in the projections as a high or low priority with an output of 0 Mt? I suggest adding a sentence explicitly stating whether closure is considered in the modeling and what it entails for the overlay with priority areas.

S&P data on ore and concentrate production tonnages is very incomplete, which I assume are crucial for estimating remaining reserves and their grade at existing mines. It would be helpful to have a clear overview of the quality of the S&P data used as input to PEMMSS and to flag the various projects accordingly (low, medium, high quality data).

L375-376: Related to the above, where are the results of the validated resource tonnage data against the data from Mervine et al.? They are not referenced with a figure or table here; I would definitely include that.

Reviewer #4 (Remarks to the Author):

This is an excellent manuscript that addresses an important and understudied aspect of moving towards a net-zero future, the adverse biodiversity impacts of a growing nickel supply required to power a net-zero future. The authors explore this through the new PEMMSS model, a model that is firmly gounded in reliable and sound data sources. By combining the IEA’s global decarbonization scenarios with global conservation targets the modeling assumptions are reasonable and rooted in the latest research and policy discussions. The manuscript is also important by highlighting Indonesia’s outsized role in the current nickel supply, a recent phenomenon with massive adverse environmental and biodiversity impacts. The

methodology is sound, includes reasonable and relevant scenarios, and good sensitivity analyses. The authors may want to explain a bit more why the possibility to supply nickel from secondary (recycled) sources is so limited. The one sentence provided (line 115) may not be easily understood by readers that don't follow critical material discussions closely. This could include a reminder of the importance of recycling (and circular economy, design for recycling). With this, I consider the manuscript ready for publication.

Minor comment:
Line 293: I'd suggest slightly rephrasing this sentence, adding the caveat that "current" decarbonization pathways require lots of nickel, while technology change might mitigate this high demand.

********************END********************

Version 1:

Decision Letter:

5th March 2026

Dear Dr. Hyman,

Thank you for submitting your revised manuscript "Growing nickel supply from the tropics threatens priority conservation areas" (NATECOLEVOL-25113888A). It has now been seen again by three of the original reviewers, whose comments are below. The reviewers find that the paper has improved in revision, and therefore we'll be happy in principle to publish it in Nature Ecology & Evolution, pending minor revisions to comply with our editorial and formatting guidelines.

If the current version of your manuscript is in a PDF format, please email us a copy of the file in an editable format (Microsoft Word or LaTex)-- we can not proceed with PDFs at this stage.

If you have not done so already, please ensure that you also email us a completed copy of the Reporting summary :

Reporting summary: https://www.nature.com/documents/nr-reporting-summary.pdf

We are now performing detailed checks on your paper and will send you a checklist detailing our editorial and formatting requirements in about a week. Please do not upload the final materials and make any revisions until you receive this additional information from us.

Thank you again for your interest in Nature Ecology & Evolution. Please do not hesitate to contact me if you have any questions.

[redacted]

Reviewer #1 (Remarks to the Author):

The detailed revisions and responses to comments are adequate in my view to move this paper to publication.

Reviewer #3 (Remarks to the Author):

I commend the authors for a very thorough revision of my comments. Especially the added detail on discussions about post-mining impacts, which addresses uncertainties regarding the underlying biodiversity data and the radii used to map threats. I have no further comments, and I strongly hope to see this paper published.

Reviewer #4 (Remarks to the Author):

I thank the authors for their very careful and comprehensive response to my and the other reviewers' comments, which further improved the clarity and depth of the manuscript. With this revision I consider the manuscript ready for publication.

Open Access This Peer Review File is licensed under a Creative Commons Attribution 4.0 International License, which permits use, sharing, adaptation, distribution and reproduction in any medium or format, as long as you give appropriate credit to the original author(s) and the source, provide a link to the Creative Commons license, and indicate if changes were made.
In cases where reviewers are anonymous, credit should be given to 'Anonymous Referee' and the source.
The images or other third party material in this Peer Review File are included in the article's Creative Commons license, unless indicated otherwise in a credit line to the material. If material is not included in the article's Creative Commons license and your intended use is not permitted by statutory regulation or exceeds the permitted use, you will need to obtain permission directly from the copyright holder.
To view a copy of this license, visit https://creativecommons.org/licenses/by/4.0/

Response to Reviewers (NATECOLEVOL-25113888-T)

Growing nickel supply from the tropics threatens priority conservation areas

Jayden Hyman[1,2], Laura J. Sonter[1,2,3,4], Eve McDonald-Madden[1,2], James E. M. Watson[1,2], Evelyn M. Mervine[1], Joseph W. Bull[5], Chloe Dawson[1,2], Thomas J. Lloyd[1,2,6], Sebastian Luckeneder[7], Martine Maron[1,2], Bernardo Mendonca Severiano[8], Sarah Raymond[5], Thomas A. Schlacher[9], Rachakonda Sreekar[1,2], Rick K. Valenta[3], Piero Visconti[10], Tim T. Werner[11], Stephen A. Northey[8]

1. School of the Environment, The University of Queensland, Brisbane, Queensland, Australia
2. Centre for Biodiversity and Conservation Science, The University of Queensland, Brisbane, Queensland, Australia
3. Sustainable Minerals Institute, The University of Queensland, Brisbane, Queensland, Australia
4. The Biodiversity Consultancy, Cambridge, United Kingdom
5. Department of Biology, University of Oxford, United Kingdom
6. College of Science & Engineering, James Cook University, Townsville, Queensland, Australia
7. Stockholm Resilience Centre, Stockholm University, Stockholm, Sweden
8. Institute for Sustainable Futures, University of Technology Sydney, Ultimo, New South Wales, Australia
9. School of Science, Technology and Engineering, University of the Sunshine Coast, Maroochydore, Queensland, Australia
10. Biodiversity, Ecology, and Conservation Group, Biodiversity and Natural Resources Program, International Institute for Applied Systems Analysis, Laxenburg, Austria
11. School of Geography, Earth and Atmospheric Sciences, The University of Melbourne, Carlton, Victoria, Australia

* Corresponding author: j.hyman@uq.edu.au

Reviewer #1 (Remarks to the Author):

This is an excellent article that uses specific demand scenarios for a critical metal for the green transition and links that to key economically viable reserves in the tropics. Such an approach can assist in considering broader prioritization of supply sources be subsequently using tools such as Life Cycle Analysis.

Response 1.1: We thank the reviewer for their interest in our work, the positive review, and constructive comments. We agree that our work could help in prioritising supply sources and informing LCA.

The rise in supply from lateritic sources rather than magmatic sulfides has been under-studied. There are three areas where revisions would be needed before publication:

a) The rise in supply scenarios for magmatic sulfides closer to 2050 in figure 1b needs to be better explained. There are major exploration projects underway and also new techniques to harness lower grade sulfide ores. The authors should consult with the International Nickel Study Group in Portugal and the Nickel Institute in this regard to test the validity of their projections.

Response 1.2: The rise in magmatic sulphide production closer to 2050 in Fig. 1b reflects the deposit characteristics in our dataset. Laterite deposits typically have higher ore tonnage and nickel grade combinations (sulphides often have high grade or high tonnage, but rarely both, see Extended Data Fig. 2), making them more competitive in our primary scenario (APS-T) and across value models (Extended Data Fig. 4) and thus prioritised earlier in our simulations. Additionally, some sulphide projects have a lower chance of entering production over the next decade, since they are in early exploration, and greater lead times are captured through the PEMMSS model's development probability feature.

To address this point about emerging technologies for harnessing low-grade ores, we conducted an additional sensitivity analysis that prioritises deposits based on ore tonnage alone, rather than our other value models which incorporate grade (see APS-T-T in Extended Data Fig. 4c and Extended Data Table 1). This represents a theoretical scenario where grade effectively plays no role in a projects value, and the largest deposits are prioritised. While this scenario leads to slightly greater cumulative production from magmatic sulfides by 2050, laterites still dominate supply over the study period due to the scale of known laterite deposits. We amended Extended Data Table 1 and Extended Data Fig. 4 to include this scenario.

We have also amended this sentence in the methods:

> "We also tested the sensitivity of our results to alternative value models, including the contained nickel in a deposit ($C = T \cdot G$), a deposit’s ore grade alone (G), and ore tonnage alone (T)."

We appreciate the reviewer's suggestion to consult with the International Nickel Study Group (INSG) and Nickel Institute. Our deposit-level resource data are sourced from the S&P Capital IQ Pro database, which compiles data from company reports, including those prepared under internationally recognised reporting standards (JORC Code, NI 43-101). We acknowledge that

consulting industry organisations could provide valuable context and proprietary data on supply dynamics and help validate our scenario modelling, noting that several of the coauthors of our study have worked closely with these groups in the past. We believe that our work provides robust and valuable insights without the need for direct input from industry organisations but nonetheless provides a modelling framework for these organisations to understand the threats of future supply for areas important for biodiversity and carbon conservation. We plan to engage with these groups following publication to explore how our PEMMSS modelling framework could inform industry discussions on supply prioritisation.

b) The authors have too casually dismissed the secondary source projections based on an assumption of current supply being locked in infrastructure. However, nickel has been used in steel production for over a century and there are indeed many supply sources which hitherto have perhaps been neglected. For example, the "ghost cities of China" and other sources of nickel in ageing infrastructure from urban mining needs to be acknowledged. Refer to the following study https://www.nature.com/articles/s41467-020-15246-4 *as a starting point.*

Response 1.3: We appreciate the reviewer's concern regarding secondary supply and urban mining potential. The paper cited by the reviewer (Zeng et al. 2020) maps the generation of anthropogenic minerals across three product groups in China (waste electronics, end-of-life vehicles, and waste wires and cables) but does not include nickel among the 23 materials analysed. However, an earlier study by the same lead author (Zeng et al. 2018) directly examined China's nickel supply sustainability, finding that even under very high end-of-life recycling rates (>80%), secondary supply falls short of what would be needed to meet domestic demand, underscoring the importance of primary supply in the near term.

As stated in our manuscript, "secondary supply will become increasingly important by mid-century to reduce reliance on mined nickel but is projected to play a minor role in the near-term (~3% share by 2030, ~10% share by 2040), constrained by the rate at which products reach their end of life for recycling." The key constraint is timing. Reck et al. (2008) found that nickel products have long lifetimes – particularly stainless steel in buildings and infrastructure (~50 years) – meaning current stocks will only become available for recycling in several decades. The reviewer's example of "ghost cities" illustrates this point. Although there is a lot of nickel locked up in this infrastructure constructed in the 2000s and 2010s, they will not reach end-of-life until the 2050s or 2060s, after our study period. While early demolition for scrap recovery is an interesting idea, it is unlikely to contribute meaningfully within our study period given the associated costs and China's existing capital investments focusing on Indonesian laterite production. If our model timeline was extended to 2100 and beyond, nickel that is locked up in recently constructed but relatively underutilised infrastructure would certainly be an important factor to consider. Based on the reviewer's feedback, we have revised our discussion on secondary supply below:

> "Secondary supply (nickel recovered from recycling) will become increasingly important by mid-century to reduce reliance on mined nickel but is projected to play a minor role in the near-term (~2% in 2024, ~1.5–3% by 2030, ~5–10% by 2040 across STEPS and APS scenarios[12,30]), constrained by the rate at which products reach their end of life for recycling[12]. Nickel is predominantly used in long-lived applications, including stainless

> steel in buildings and infrastructure (~50 year lifespan) and industrial machinery (~25 years)[31,32]. The amount of nickel available for recycling today reflects the lower production volumes of past decades[6], and the in-use stocks being accumulated now may only become available for recycling at significant volumes by mid-century[12,31,33]. Current end-of-life recycling rates fall far short of what is needed to offset primary supply requirements, and scaling secondary supply will require improvements in collection systems, urban mining, and design for recyclability[33,34]. Even with increasing adoption of low- or no-nickel battery chemistries[12,15], total demand is projected to grow substantially across all of the International Energy Agency's scenarios due to nickel's importance for stainless steel, renewables, and other applications (Extended Data Fig. 1)."

c)The authors should also acknowledge that some of the arguments for mining pertain to the economic development impact on the extractive countries. In particular Indonesia and the Philippines which see this as a major source of income for national accounts. Some discussion of alternative development paths for these countries should also be noted (they clearly have potential for various service sector economies as well).

Response 1.4: We thank the reviewer for highlighting the economic development dimensions of nickel mining. We acknowledge that Indonesia and the Philippines view nickel as a strategic resource for national development, and that any discussion of supply constraints must consider these economic realities. We have therefore amended our discussion to acknowledge this:

> "…without a viable alternative resource stream. Furthermore, nickel mining offers resource-rich developing countries a vital source of income and employment, although overreliance can leave local communities vulnerable to market disruptions. Ensuring the adoption of standards for ecologically responsible mining..."

With these changes this article would be ready for publication

Response 1.5: We thank the reviewer for their constructive suggestions and the recommendation for publication.

Reviewer #2 (Remarks to the Author):

This is a very well-written, comprehensive and topical manuscript outlining the potential impacts of nickel mining on priority conservation areas. The modelling conducted considers a wide variety of issues related to nickel sulphide, laterite and deep sea nodule mining and presents scenarios for these related to biodiversity for the coming decades. Overall, the manuscript is excellent and I recommend acceptance following very minor revision and consideration of the following comments:

Response 2.1: We thank the reviewer for their positive comments and recommendation for acceptance following very minor revision. We have incorporated all their constructive suggestions. Please see our responses below.

Line 114. - 'an estimated 55 to 96 new nickel mines starting produciton by 2050'. How close to reality are these estimates, given the length of time it can take (10-20 years) to get a mine up and running?

Response 2.2: The estimate of new mines starting production by 2050 varies across modelled scenarios. Our scenarios tend to prioritise larger, higher-grade deposits first, so fewer new mines are required in the near-term to meet demand, i.e. a median of 21 start production by 2040 under the Announced Pledges Scenario (see Extended Data Fig. 5). This rate (~1–2 new mines per year) aligns well with rates of historical mine development. For example, Heijlen et al. (2021) found that 30 nickel mines were started between 2000 and 2020 (~1.5 per year). In the terrestrial-only scenario, beyond 2040, large, high-grade mines become depleted, and new, smaller-tonnage and/or lower-grade deposits are required to meet demand. The 55–96 mines starting between 2025 and 2050 under APS-T (~2–4 per year) represents approximately double the historical rate (and this could be seen as consistent with the projected doubling of global nickel demand). The reviewer correctly notes that mine development can take 10–20 years. Heijlen et al. (2021) found that while overall lead times have increased to a median of 12 years, the time between feasibility to production has remained constant at ~4 years. Several projects in our dataset are already at the more advanced stages of development while others are still in early exploration and it is reasonable to assume several projects may move from early exploration to production within the 25 year development window of our model. As described in our Methods, our scenarios capture uncertainty regarding which specific mines enter production through the PEMMSS model development probability feature, which is informed by their current development stage. Here, we would also like to emphasise that our analysis focuses on whether development of known resources could meet projected demand, not whether specific projects are likely to be developed given permitting, financing, and other project-specific constraints.

Line 115 - Please define what you mean by 'secondary supply'. If this does not include tailings recycling then please add the estimate of Ni recovery from this secondary supply to your estimates in these sentences. If the estimates for Ni recovery from tailings is large, please state how this would affect your modelling and conclusions.

Response 2.3: We have now amended the following paragraph to include a brief definition of secondary supply in the first sentence:

> "Secondary supply (nickel recovered from recycling) will become increasingly important by mid-century to reduce reliance on mined nickel but is projected to play a minor role in the near-term (~2% in 2024, ~1.5–3% by 2030, ~5–10% by 2040 across STEPS and APS scenarios[12,30]), constrained by the rate at which products reach their end of life for recycling[12]. Nickel is predominantly used in long-lived applications, including stainless steel in buildings and infrastructure (~50 year lifespan) and industrial machinery (~25 years)[31,32]. The amount of nickel available for recycling today reflects the lower production volumes of past decades[6], and the in-use stocks being accumulated now may only become available for recycling at significant volumes by mid-century[12,31,33]. Current end-of-life recycling rates fall far short of what is needed to offset primary supply requirements, and scaling secondary supply will require improvements in collection systems, urban mining, and design for recyclability[33,34]. Even with increasing adoption of low- or no-nickel battery chemistries[12,15], total demand is projected to grow substantially across all of the International Energy Agency's scenarios due to nickel's importance for stainless steel, renewables, and other applications (Extended Data Fig. 1)."

We note that Nickel recovered from the tailings of former mining projects was not considered in our study. Mudd and Jowitt (2022) classify tailings deposits as a distinct resource category and show that nickel contained in code-compliant and non-code-compliant tailings resources represents only 0.49 Mt Ni (~0.14% of global nickel resources), and all have less than 0.5 % Ni ore grade. Given this negligible contribution, including tailings deposits from current or former mining operations would not materially affect our modelling results or conclusions. Moreover, these tailings deposits are mostly in Australia, where the majority of existing mines have recently entered care and maintenance. We have added the following sentence to the 'Model limitations' section:

> "Reprocessing of tailings deposits of former projects was not considered in our study. These deposits represent an estimated 0.49 Mt of nickel[6], and hence their inclusion would not materially affect our modelling results."

Line 182. Please change 'tailings dams' to 'tailings storage facility' (and then change the 'be stored in' to something like 'be deposited in'. Also - what is your reference for the 'billions of tones of waste'? Has this number been properly modelled?

Response 2.4: Agreed. We have amended the sentence as requested:

> "This requires mining and processing an estimated 7.4 to 8.4 Gt of ore between 2025 and 2050 upstream of global strongholds of marine biodiversity, generating billions of tonnes of waste that would need to be deposited in tailings storage facilities (Extended Data Fig. 7c)."

In the sentence above, we have moved the cross reference to Extended Data Fig. 7c to the end to clarify that this is a result of our modelling. This figure supports the billions of tonnes of waste result. Since average mined ore grades are around 1% of the mass of ore mined (see Extended Data Fig. 5b), the vast majority of the processed ore mass (estimated at 7.4 to 8.4 billion tonnes) becomes tailings (i.e. the other 99%).

Line 184. Please change' tailings dam failures' to 'tailings storage facility failures'. Also change 'pollution' to 'contamination' (pollution is contamination that has a demonstrated negative effect on human health).

Response 2.5: Agreed. We have amended the sentence for consistency:

> "Throughout these mining operations, and even following their closure, ongoing sediment and heavy metal contamination, the potential for catastrophic tailings storage facility failures, and practices of marine or riverine tailings disposal, could pose substantial threats..."

Reviewer #2 (Remarks on code availability): I am not familiar enough with modelling to run and understand codes like this.

Reviewer #3 (Remarks to the Author):

Hyman et al. present a prospective analysis of potential conflicts between Nickel mining and priority conservation areas, including both terrestrial and deep-sea mining. I greatly enjoyed the paper, which is very clearly written and among the first analyses to compare potential impacts on biodiversity from terrestrial and deep-sea sources, even if in a simplified overlay. I have identified below a number of issues that I hope will help strengthen the paper before acceptance.

Response 3.1: We thank the reviewer for their thoughtful and constructive feedback. We are happy to hear that they enjoyed reading the paper, and we appreciate the recognition of its novelty. We have incorporated all the reviewer's suggestions in our revised manuscript. Please see our responses below.

Main comments

After a mine closes, impacts can vary significantly depending on the country and type of mine. Moreover, once a deposit is fully exploited, the lasting impacts on biodiversity will differ greatly between terrestrial and marine mines. I suggest the authors expand the paragraph starting at L141 to include a more in-depth discussion of their findings in the context of post-mining impacts across different ecosystems.

Response 3.2: Agreed. We have now added the following dedicated paragraph following the discussion of post-mining threats to terrestrial and marine priorities (rather than L141, as this section focuses on supply distribution).

> "Mining activities affect terrestrial, freshwater, and marine biodiversity through numerous impact pathways and mechanisms, with aquatic pollution identified as a pervasive pressure[38]. Strip mining in tropical forests exposes bare ground and waste rock to erosion (Fig. 2), and heavy rainfall mobilises metal-contaminated sediments that can travel tens to hundreds of kilometres downstream, persisting in freshwater systems for centuries or polluting nearshore marine environments[36,37,39]. While the absence of a globally consistent conservation priority layer precluded modelling of threats to freshwater biodiversity, recent assessments show that metal mining is degrading riverine ecosystems worldwide through increased sediment loads and long-distance contaminant transport derived from active and inactive mine sites[36], in addition to artisanal and small-scale operations[40]. In Indonesia, current regulations do not require restoration to pre-mining conditions – emphasising alternative post-mining land uses – and progressive rehabilitation remains rare, despite ongoing deforestation within mining concessions[41]. Without effective rehabilitation, cross-realm threats can persist after mining operations cease, particularly when sites are abandoned. Even with active restoration, forest degradation and biomass carbon loss can often only be partially reversed over reasonable timescales[42,43]. Given the potential growth of nickel mining in highly biodiverse coastal ecosystems in the tropics, subnational assessments of cross-realm threats are a critical research priority."

Similarly, we have also added the following on post-mining impacts of deep-sea mining of polymetallic nodules in the section on the implications of marine resource development:

> "The abyssal seafloor of the CCZ (~4–6 km depth) is a food-limited, low-biomass environment hosting more than 6,000–8,000 metazoan species – primarily small worms and crustaceans from the phyla Annelida, Nematoda, and Arthropoda[25,46]. The lasting biodiversity impacts of deep-sea mining depend largely on the technology used. Modern systems disturb the top 3–5 cm of sediment, whereas long-term disturbance studies focus on systems that were more invasive, such as the Ocean Minerals Company (OMCO) mining test in 1979, which created 20–80 cm deep furrows[25,47–49]. The OMCO test caused long-lasting impacts on invertebrate communities living on the seafloor, while sediment-dwelling organisms and microbial communities had similar densities to undisturbed control sites[48]. Current evidence suggests impacts on nodule-dependent taxa are expected to be long-lasting across contract areas, and recovery will depend on whether there is sufficient hard substrate left unmined to support recolonisation[25,48]. Two months following a recent industrial trial in 2022, sediment-dwelling macrofauna were approximately two-thirds of their baseline density and diversity in directly mined areas[47]. In nearby areas affected by sediment plumes, neither short-term nor long-term studies found significant decreases in faunal abundance compared to control sites[47,48]. We stress that any future polymetallic nodule mining requires effective and evidence-based environmental management to mitigate adverse effects on deep-sea biodiversity and ecosystem services[48,50], and that achieving such outcomes may present a significant challenge[51,52]. We also note that while the development of deep-sea projects may supplement a portion of supply from terrestrial sources, this does not guarantee a wholesale reduction in terrestrial impacts, as existing operators may still fail to scale back land clearing efforts, manage downstream pollution, and implement post-mine rehabilitation."

While I appreciated the detailed discussion on the limitations of the PEMMSS approach, I missed context regarding the limitations of the biodiversity data from Jung et al. and how these limitations influence the results presented in this manuscript. I encourage the authors to discuss potential biases and the representativeness of the dataset used to identify priority conservation areas. Additionally, it would be helpful to clarify how these factors relate to existing protected areas and other commonly used metrics, such as threat status, and to what extent freshwater ecosystems are represented by these layers.

Response 3.3: We thank the reviewer for raising this important point. Please refer to this revised sentence in our methods:

> "We note that although both terrestrial and marine priority maps have coarse resolution, they use the best available data on species distribution, with particular attention to biases introduced by incomplete knowledge on distribution of species in selected global regions (the so-called Wallacean shortfall) and are therefore considered fit-for-purpose for global-scale analyses."

We have also now expanded our discussion of the strengths and limitations of the Jung et al. (2021) priority maps in the "Model limitations":

> " The terrestrial conservation priority maps used in our analysis represent the most comprehensive global integration of biodiversity and carbon data to date, including all extant terrestrial vertebrates (5,685 mammals, 6,660 amphibians, 10,953 birds, and 10,585 reptiles), a representative subset of all accepted vascular plant species (~41%, 193,954 species), and the best available maps of above-ground and below-ground biomass carbon and vulnerable soil carbon[22]. However, the study notably lacks freshwater, soil, and invertebrate species, for which data are currently insufficient for global-scale analyses. Similarly, the ocean priority maps represent the most comprehensive global assessment of priorities for conserving marine biodiversity, integrating multiple data sources[29]. While each study employs realm-specific methodologies for its global optimisation, both consider species threat status, integrate existing protected areas, and conduct extensive uncertainty analyses[22,29]. Although any one analysis has its limitations, multiple global-scale studies confirm that the land and coastal ecosystems of top nickel producers – including Indonesia (Wallacea), the Philippines, and New Caledonia – are global hotspots of terrestrial and marine species endemism[68–70]. We consider the maps used in our analysis appropriate for global-scale analyses of mining threats, but emphasise that focused subnational assessments would be required to evaluate impacts on individual species and ecosystems."

Many of the marine impacts rely on the assumption that mines within 50 km of coastlines contribute to marine impacts. First, for land mines, the radius used to identify overlap with conservation priority areas is 1 to 10 km, which is inconsistent with the 50 km radius. Second, what is the rationale behind the impact mechanisms for land or coastal mines affecting marine biodiversity? It is unclear how the link to the 50 km radius of indirect impacts from Sonter et al. (which remains, in any case, very arbitrary and unverified) indicates how a mine connects land to the coast. I encourage the authors to better support this assumption by explaining the impact pathways that justify using a 50 km radius for land-to-marine impacts. If pollution is the main concern, they could consider using drainage lines with a justified distance threshold (since not all mines within 50 km of the coast might connect to it, depending on local terrain). If the authors decide to maintain a buffer approach, I recommend at least conducting a sensitivity analysis using 10 km and 50 km, as in Sonter et al. (2020).

Response 3.4: We thank the reviewer for these thoughtful comments on our methodological choices regarding the 50 km buffer threshold, which we address in detail below. Our 50 km threshold is justified on multiple grounds and represents a conservative choice relative to documented direct and indirect mining impacts in global-scale analyses. First, our 50 km buffer is constrained by the spatial resolution of the underlying marine conservation priority layer from Sala et al. (2021), which uses ~50 km grid cells (the best resolution available). A 50 km buffer guarantees that at least one raster cell is sampled. Although the ~50 km resolution of the marine priority data precludes a 10 km sensitivity analysis, we have now added Extended Data Fig. 7d to characterise the spatial distribution of deposits relative to coastlines. This figure shows that an estimated ~170 Mt Ni occurs within 50 km of coastlines (~88 Mt Ni within 10 km; ~82 Mt Ni

between 10–50 km), compared to ~68 Mt Ni beyond 50 km. Adopting a 10 km threshold would reclassify ~82 Mt Ni as posing no threat to marine biodiversity, despite evidence that mining impacts extend well beyond 10 km. Given the global scale of our analysis and that most coastal waters adjacent to laterite deposits within 50 km of coastlines rank in the top 10% of marine biodiversity priorities, we consider the 50 km threshold appropriately conservative. We believe the burden of evidence lies with demonstrating that indirect and downstream effects of laterite mining can be contained within 10 km – evidence that does not currently exist. The diverse pathways by which mining activities impact biodiversity is emerging as a critical area of research (see Barbarossa et al.[39], which is cited in our revised manuscript as per Response 3.2). We emphasise that subnational assessments are critical for evaluating mine-specific impact pathways.

While drainage-line analysis would provide finer spatial precision, implementing this approach at a global scale would introduce substantial methodological complexity without materially altering our core findings, given that most laterite deposits occur on small tropical islands where catchments drain directly to the coast. As shown in our new figure (Extended Data Fig. 7d), a significant portion of nickel in known laterite deposits is within kilometers of coastal ecosystems (i.e. often on steep islands in the Coral Triangle). We have added the following sentence to the main text:

> "An estimated 170 Mt of nickel contained in laterite deposits occurs within 50 km of coastlines (88 Mt Ni within 10 km; 82 Mt Ni between 10–50 km), compared to just 2 Mt for magmatic sulphide deposits, which are predominantly located greater than 50 km inland (Extended Data Fig. 7d)."

Where mines drain to closed basins rather than coastal waters, the focus should shift to freshwater contamination – a point we have noted in our revised discussion of cross-realm threats.

We have added the following justification to the Model Limitations section, drawing on literature documenting direct and indirect mining impacts:

> "While significant progress has been made in recent years to map the direct footprint of global mining[56], substantial uncertainty remains regarding indirect and offsite impacts. Available evidence suggests mining poses threats to ecosystems well beyond site boundaries[38,41]. For example, Sonter et al.[17] show that mining-induced deforestation in the Brazilian Amazon can extend up to 70 km from lease boundaries, and Macklin et al.[36] highlight that sediment-associated contaminants can travel 10–100 km downstream. Nickel laterite operations are often expansive and require extensive coastal infrastructure that directly affects local marine environments, exemplified by the construction of major coastal industrial parks in Indonesia in the past decade[20]. In our study, we applied a 50 km buffer to identify deposits with the potential to threaten marine conservation priorities (Fig. 3; Extended Data Fig. 7; Extended Data Fig. 8). This threshold is constrained by the ~50 km resolution of available marine priority data[29], ensuring at least one raster cell is sampled, but is nonetheless consistent with buffers used in recent global mining threat assessments[17]. While global mine-by-mine depletion

is explicitly represented by our supply scenarios, we do not model the persistence or reduction of threats to conservation priorities following mine closure. Ultimately, global prioritisation studies such as ours should be used to identify areas where regional and local impact assessments may be particularly effective for informing conservation action."

With permission from the photographer Tom Hegen, we have also added a new main text figure (Fig. 2 – adjusting subsequent figure numbers), depicting a coastal laterite mining operation in Sulawesi, Indonesia, with sediment-laden water near a small village, providing a useful visual reference for readers.

Minor comments

L158: Depending on the screen, the 100% shade is barely visible. Please increase the contrast or use contours. The same applies to Figure 2.

Response 3.5: The outer shade represents 100% of simulations, i.e. the top 5% and bottom 5% are not covered by the darker shade. We agree that it can be difficult to see and may make it harder to interpret the graphs. We have decided to remove the outer shade, as we did not deem it necessary for reporting results (which we primarily report 5th to 95th percentiles throughout). This also ensures consistency between line and bar charts. We have updated all associated figures.

L152-163: It is unclear what the "-T" represents in the scenario acronyms just by looking at the figure and caption. I need to get to the Supplement to learn that it refers to terrestrial. Please spell it out in the caption.

Response 3.6: We have updated the captions to explain that -T means terrestrial only scenario at each relevant caption location:

> "The line charts (a and b) show the median annual terrestrial nickel production under the Announced Pledges Scenario (APS-T, where T denotes terrestrial resources only) across model simulations..."

L141-143: How did the authors handle mine closure in their impact assessment modeling? If a mine closes, does the impact continue? In that case, is the mine still included in the projections as a high or low priority with an output of 0 Mt? I suggest adding a sentence explicitly stating whether closure is considered in the modeling and what it entails for the overlay with priority areas.

Response 3.7: Our modelling considers a mine that is depleted as still poseing a threat to biodiversity throughout our 25 year window. Please note, we do not consider our assessment as an "impact assessment", but rather a global threat assessment based on the proximity of deposits to conservation priorities. We have added a sentence to emphasise that our global analysis can directly inform where impact assessments should be prioritised:

> "While global mine-by-mine depletion is explicitly represented by our supply scenarios, we do not model the persistence or reduction of threats to conservation priorities

> following mine closure. Ultimately, global prioritisation studies such as ours should be used to identify areas where regional and local impact assessments may be particularly effective for informing conservation action."

S&P data on ore and concentrate production tonnages is very incomplete, which I assume are crucial for estimating remaining reserves and their grade at existing mines. It would be helpful to have a clear overview of the quality of the S&P data used as input to PEMMSS and to flag the various projects accordingly (low, medium, high quality data).

Response 3.8: We do not use ore or concentrate production tonnages from S&P, as these data are indeed very incomplete. Instead, we use the most recent reported remaining resources (including reserves) for existing mines (and undeveloped deposits) as inputs to the PEMMSS model, where we then estimate production capacity using Taylor's rule (see Methods and Extended Data Fig. 3). This approach avoids reliance on incomplete production data and instead leverages resource estimates that are more consistently reported. We do note the limitations of S&P in our study, while acknowledging it as the best available dataset. For example:

> " However, currently half of global mining areas lack basic production information necessary for independent impact assessments. This is particularly prevalent in Russia, China, Indonesia, and Brazil[56]…"

> "The S&P Database may contain incomplete data, particularly relating to production, due to issues of transparency affecting large portions of the mining industry..."

We have also now added the following sentences to the Model limitations section:

> "Resource reporting quality varies by jurisdiction[56], and established mining regions may have well-documented resources that are unlikely to be developed due to factors beyond the scope of our model (e.g., permitting constraints, community opposition, or insufficient processing capacity), potentially leading to overestimates of production. Conversely, emerging frontiers such as Indonesia may have limited or delayed reporting that does not capture ongoing exploration, potentially leading to underestimates of production."

Ranking the quality of available data would be difficult to do in an objective way, as reporting standards vary by jurisdiction and over time. We believe our approach of using resource estimates (which are more consistently reported under international standards such as JORC and NI 43-101) rather than production data, combined with transparent acknowledgement of data limitations, provides a robust and consistent foundation for our global-scale analysis.

L375-376: Related to the above, where are the results of the validated resource tonnage data against the data from Mervine et al.? They are not referenced with a figure or table here; I would definitely include that.

Response 3.9: Agreed. We have added a new panel (Extended Data Fig. 2b) comparing contained nickel by country and deposit type in our study with estimates from Mervine et al. (2025), who report data up to 2020. The comparison shows general agreement, with increases

in contained nickel in our dataset reflecting exploration and improved reporting in recent years, particularly in Indonesia, Canada, and New Caledonia.

Reviewer #4 (Remarks to the Author):

This is an excellent manuscript that addresses an important and understudied aspect of moving towards a net-zero future, the adverse biodiversity impacts of a growing nickel supply required to power a net-zero future. The authors explore this through the new PEMMSS model, a model that is firmly gounded in reliable and sound data sources. By combining the IEA's global decarbonization scenarios with global conservation targets the modeling assumptions are reasonable and rooted in the latest research and policy discussions. The manuscript is also important by highlighting Indonesia's outsized role in the current nickel supply, a recent phenomenon with massive adverse environmental and biodiversity impacts. The methodology is sound, includes reasonable and relevant scenarios, and good sensitivity analyses. The authors may want to explain a bit more why the possibility to supply nickel from secondary (recycled) sources is so limited. The one sentence provided (line 115) may not be easily understood by readers that don't follow critical material discussions closely. This could include a reminder of the importance of recycling (and circular economy, design for recycling). With this, I consider the manuscript ready for publication.

Response 4.1: We thank the reviewer for their positive review and recommendation for publication. We appreciate the suggestion to expand our explanation of secondary supply constraints. We have now amended the following paragraph on why secondary supply is limited:

> "Secondary supply (nickel recovered from recycling) will become increasingly important by mid-century to reduce reliance on mined nickel but is projected to play a minor role in the near-term (~2% in 2024, ~1.5–3% by 2030, ~5–10% by 2040 across STEPS and APS scenarios[12,30]), constrained by the rate at which products reach their end of life for recycling[12]. Nickel is predominantly used in long-lived applications, including stainless steel in buildings and infrastructure (~50 year lifespan) and industrial machinery (~25 years)[31,32]. The amount of nickel available for recycling today reflects the lower production volumes of past decades[6], and the in-use stocks being accumulated now may only become available for recycling at significant volumes by mid-century[12,31,33]. Current end-of-life recycling rates fall far short of what is needed to offset primary supply requirements, and scaling secondary supply will require improvements in collection systems, urban mining, and design for recyclability[33,34]. Even with increasing adoption of low- or no-nickel battery chemistries[12,15], total demand is projected to grow substantially across all of the International Energy Agency's scenarios due to nickel's importance for stainless steel, renewables, and other applications (Extended Data Fig. 1)."

Minor comment:
Line 293: I'd suggest slightly rephrasing this sentence, adding the caveat that "current" decarbonization pathways require lots of nickel, while technology change might mitigate this high demand.

Response 4.2: Agreed. We have amended the sentence:

"Current pathways to decarbonisation require a substantial increase in nickel supply over the coming decades, although the pace of demand growth will depend on policy ambitions and technological developments."

Response to Reviewers

Second round

Reviewer #1 (Remarks to the Author):

The detailed revisions and responses to comments are adequate in my view to move this paper to publication.

Reviewer #2 (Remarks to the Author):

None

Reviewer #3 (Remarks to the Author):

I commend the authors for a very thorough revision of my comments. Especially the added detail on discussions about post-mining impacts, which addresses uncertainties regarding the underlying biodiversity data and the radii used to map threats. I have no further comments, and I strongly hope to see this paper published.

Reviewer #4 (Remarks to the Author):

I thank the authors for their very careful and comprehensive response to my and the other reviewers' comments, which further improved the clarity and depth of the manuscript. With this revision I consider the manuscript ready for publication.

First round

Reviewer #1 (Remarks to the Author):

This is an excellent article that uses specific demand scenarios for a critical metal for the green transition and links that to key economically viable reserves in the tropics. Such an approach can assist in considering broader prioritization of supply sources be subsequently using tools such as Life Cycle Analysis.

Response 1.1: We thank the reviewer for their interest in our work, the positive review, and constructive comments. We agree that our work could help in prioritising supply sources and informing LCA.

The rise in supply from lateritic sources rather than magmatic sulfides has been under-studied. There are three areas where revisions would be needed before publication:

a) The rise in supply scenarios for magmatic sulfides closer to 2050 in figure 1b needs to be better explained. There are major exploration projects underway and also new techniques to harness lower grade sulfide ores. The authors should consult with the International Nickel Study Group in Portugal and the Nickel Institute in this regard to test the validity of their projections.

Response 1.2: The rise in magmatic sulphide production closer to 2050 in Fig. 1b reflects the deposit characteristics in our dataset. Laterite deposits typically have higher ore tonnage and nickel grade combinations (sulphides often have high grade or high tonnage, but rarely both, see Extended Data Fig. 2), making them more competitive in our primary scenario (APS-T) and across value models (Extended Data Fig. 4) and thus prioritised earlier in our simulations. Additionally, some sulphide projects have a lower chance of entering production over the next decade, since they are in early exploration, and greater lead times are captured through the PEMMSS model's development probability feature.

To address this point about emerging technologies for harnessing low-grade ores, we conducted an additional sensitivity analysis that prioritises deposits based on ore tonnage alone, rather than our other value models which incorporate grade (see APS-T-T in Extended Data Fig. 4c and Extended Data Table 1). This represents a theoretical scenario where grade effectively plays no role in a projects value, and the largest deposits are prioritised. While this scenario leads to slightly greater cumulative production from magmatic sulfides by 2050, laterites still dominate supply over the study period due to the scale of known laterite deposits. We amended Extended Data Table 1 and Extended Data Fig. 4 to include this scenario.

We have also amended this sentence in the methods:

> "We also tested the sensitivity of our results to alternative value models, including the contained nickel in a deposit ($C = T \cdot G$), a deposit's ore grade alone (G), and ore tonnage alone (T)."

We appreciate the reviewer's suggestion to consult with the International Nickel Study Group (INSG) and Nickel Institute. Our deposit-level resource data are sourced from the S&P Capital

IQ Pro database, which compiles data from company reports, including those prepared under internationally recognised reporting standards (JORC Code, NI 43-101). We acknowledge that consulting industry organisations could provide valuable context and proprietary data on supply dynamics and help validate our scenario modelling, noting that several of the coauthors of our study have worked closely with these groups in the past. We believe that our work provides robust and valuable insights without the need for direct input from industry organisations but nonetheless provides a modelling framework for these organisations to understand the threats of future supply for areas important for biodiversity and carbon conservation. We plan to engage with these groups following publication to explore how our PEMMSS modelling framework could inform industry discussions on supply prioritisation.

b) The authors have too casually dismissed the secondary source projections based on an assumption of current supply being locked in infrastructure. However, nickel has been used in steel production for over a century and there are indeed many supply sources which hitherto have perhaps been neglected. For example, the "ghost cities of China" and other sources of nickel in ageing infrastructure from urban mining needs to be acknowledged. Refer to the following study https://www.nature.com/articles/s41467-020-15246-4 *as a starting point.*

Response 1.3: We appreciate the reviewer's concern regarding secondary supply and urban mining potential. The paper cited by the reviewer (Zeng et al. 2020) maps the generation of anthropogenic minerals across three product groups in China (waste electronics, end-of-life vehicles, and waste wires and cables) but does not include nickel among the 23 materials analysed. However, an earlier study by the same lead author (Zeng et al. 2018) directly examined China's nickel supply sustainability, finding that even under very high end-of-life recycling rates (>80%), secondary supply falls short of what would be needed to meet domestic demand, underscoring the importance of primary supply in the near term.

As stated in our manuscript, "secondary supply will become increasingly important by mid-century to reduce reliance on mined nickel but is projected to play a minor role in the near-term (~3% share by 2030, ~10% share by 2040), constrained by the rate at which products reach their end of life for recycling." The key constraint is timing. Reck et al. (2008) found that nickel products have long lifetimes – particularly stainless steel in buildings and infrastructure (~50 years) – meaning current stocks will only become available for recycling in several decades. The reviewer's example of "ghost cities" illustrates this point. Although there is a lot of nickel locked up in this infrastructure constructed in the 2000s and 2010s, they will not reach end-of-life until the 2050s or 2060s, after our study period. While early demolition for scrap recovery is an interesting idea, it is unlikely to contribute meaningfully within our study period given the associated costs and China's existing capital investments focusing on Indonesian laterite production. If our model timeline was extended to 2100 and beyond, nickel that is locked up in recently constructed but relatively underutilised infrastructure would certainly be an important factor to consider. Based on the reviewer’s feedback, we have revised our discussion on secondary supply below:

> "Secondary supply (nickel recovered from recycling) will become increasingly important by mid-century to reduce reliance on mined nickel but is projected to play a minor role in the near-term (~2% in 2024, ~1.5–3% by 2030, ~5–10% by 2040 across STEPS and

> APS scenarios[3,29]), constrained by the rate at which products reach their end of life for recycling[3]. Nickel is predominantly used in long-lived applications, including stainless steel in buildings and infrastructure (~50 year lifespan) and industrial machinery (~25 years)[30,31]. The amount of nickel available for recycling today reflects the lower production volumes of past decades[26], and the in-use stocks being accumulated now may only become available for recycling at significant volumes by mid-century[3,30,32]. Current end-of-life recycling rates fall far short of what is needed to offset primary supply requirements, and scaling secondary supply will require improvements in collection systems, urban mining, and design for recyclability[32,33]. Even with increasing adoption of low- or no-nickel battery chemistries[3,7], total demand is projected to grow substantially across all of the International Energy Agency's scenarios due to nickel's importance for stainless steel, renewables, and other applications (Extended Data Fig. 1)."

c)The authors should also acknowledge that some of the arguments for mining pertain to the economic development impact on the extractive countries. In particular Indonesia and the Philippines which see this as a major source of income for national accounts. Some discussion of alternative development paths for these countries should also be noted (they clearly have potential for various service sector economies as well).

Response 1.4: We thank the reviewer for highlighting the economic development dimensions of nickel mining. We acknowledge that Indonesia and the Philippines view nickel as a strategic resource for national development, and that any discussion of supply constraints must consider these economic realities. We have therefore amended our discussion to acknowledge this:

> "…without a viable alternative resource stream. Furthermore, nickel mining offers resource-rich developing countries a vital source of income and employment, although overreliance can leave local communities vulnerable to market disruptions. Ensuring the adoption of standards for ecologically responsible mining..."

With these changes this article would be ready for publication

Response 1.5: We thank the reviewer for their constructive suggestions and the recommendation for publication.

Reviewer #2 (Remarks to the Author):

This is a very well-written, comprehensive and topical manuscript outlining the potential impacts of nickel mining on priority conservation areas. The modelling conducted considers a wide variety of issues related to nickel sulphide, laterite and deep sea nodule mining and presents scenarios for these related to biodiversity for the coming decades. Overall, the manuscript is excellent and I recommend acceptance following very minor revision and consideration of the following comments:

Response 2.1: We thank the reviewer for their positive comments and recommendation for acceptance following very minor revision. We have incorporated all their constructive suggestions. Please see our responses below.

Line 114. - 'an estimated 55 to 96 new nickel mines starting produciton by 2050'. How close to reality are these estimates, given the length of time it can take (10-20 years) to get a mine up and running?

Response 2.2: The estimate of new mines starting production by 2050 varies across modelled scenarios. Our scenarios tend to prioritise larger, higher-grade deposits first, so fewer new mines are required in the near-term to meet demand, i.e. a median of 21 start production by 2040 under the Announced Pledges Scenario (see Extended Data Fig. 5). This rate (~1–2 new mines per year) aligns well with rates of historical mine development. For example, Heijlen et al. (2021) found that 30 nickel mines were started between 2000 and 2020 (~1.5 per year). In the terrestrial-only scenario, beyond 2040, large, high-grade mines become depleted, and new, smaller-tonnage and/or lower-grade deposits are required to meet demand. The 55–96 mines starting between 2025 and 2050 under APS-T (~2–4 per year) represents approximately double the historical rate (and this could be seen as consistent with the projected doubling of global nickel demand). The reviewer correctly notes that mine development can take 10–20 years. Heijlen et al. (2021) found that while overall lead times have increased to a median of 12 years, the time between feasibility to production has remained constant at ~4 years. Several projects in our dataset are already at the more advanced stages of development while others are still in early exploration and it is reasonable to assume several projects may move from early exploration to production within the 25 year development window of our model. As described in our Methods, our scenarios capture uncertainty regarding which specific mines enter production through the PEMMSS model development probability feature, which is informed by their current development stage. Here, we would also like to emphasise that our analysis focuses on whether development of known resources could meet projected demand, not whether specific projects are likely to be developed given permitting, financing, and other project-specific constraints.

Line 115 - Please define what you mean by 'secondary supply'. If this does not include tailings recycling then please add the estimate of Ni recovery from this secondary supply to your estimates in these sentences. If the estimates for Ni recovery from tailings is large, please state how this would affect your modelling and conclusions.

Response 2.3: We have now amended the following paragraph to include a brief definition of secondary supply in the first sentence:

> "Secondary supply (nickel recovered from recycling) will become increasingly important by mid-century to reduce reliance on mined nickel but is projected to play a minor role in the near-term (~2% in 2024, ~1.5–3% by 2030, ~5–10% by 2040 across STEPS and APS scenarios[3,29]), constrained by the rate at which products reach their end of life for recycling[3]. Nickel is predominantly used in long-lived applications, including stainless steel in buildings and infrastructure (~50 year lifespan) and industrial machinery (~25 years)[30,31]. The amount of nickel available for recycling today reflects the lower production volumes of past decades[26], and the in-use stocks being accumulated now may only become available for recycling at significant volumes by mid-century[3,30,32]. Current end-of-life recycling rates fall far short of what is needed to offset primary supply requirements, and scaling secondary supply will require improvements in collection systems, urban mining, and design for recyclability[32,33]. Even with increasing adoption of low- or no-nickel battery chemistries[3,7], total demand is projected to grow substantially across all of the International Energy Agency's scenarios due to nickel's importance for stainless steel, renewables, and other applications (Extended Data Fig. 1)."

We note that Nickel recovered from the tailings of former mining projects was not considered in our study. Mudd and Jowitt (2022) classify tailings deposits as a distinct resource category and show that nickel contained in code-compliant and non-code-compliant tailings resources represents only 0.49 Mt Ni (~0.14% of global nickel resources), and all have less than 0.5 % Ni ore grade. Given this negligible contribution, including tailings deposits from current or former mining operations would not materially affect our modelling results or conclusions. Moreover, these tailings deposits are mostly in Australia, where the majority of existing mines have recently entered care and maintenance. We have added the following sentence to the 'Model limitations' section:

> "Reprocessing of tailings deposits of former projects was not considered in our study. These deposits represent an estimated 0.49 Mt of nickel[26], and hence their inclusion would not materially affect our modelling results."

Line 182. Please change 'tailings dams' to 'tailings storage facility' (and then change the 'be stored in' to something like 'be deposited in'. Also - what is your reference for the 'billions of tones of waste'? Has this number been properly modelled?

Response 2.4: Agreed. We have amended the sentence as requested:

> "This requires mining and processing an estimated 7.4 to 8.4 Gt of ore between 2025 and 2050 upstream of global strongholds of marine biodiversity, generating billions of tonnes of waste that would need to be deposited in tailings storage facilities (Extended Data Fig. 7c)."

In the sentence above, we have moved the cross reference to Extended Data Fig. 7c to the end to clarify that this is a result of our modelling. This figure supports the billions of tonnes of waste result. Since average mined ore grades are around 1% of the mass of ore mined (see Extended Data Fig. 5b), the vast majority of the processed ore mass (estimated at 7.4 to 8.4 billion tonnes) becomes tailings (i.e. the other 99%).

Line 184. Please change' tailings dam failures' to 'tailings storage facility failures'. Also change 'pollution' to 'contamination' (pollution is contamination that has a demonstrated negative effect on human health).

Response 2.5: Agreed. We have amended the sentence for consistency:

> "Throughout these mining operations, and even following their closure, ongoing sediment and heavy metal contamination, the potential for catastrophic tailings storage facility failures, and practices of marine or riverine tailings disposal, could pose substantial threats..."

Reviewer #2 (Remarks on code availability): I am not familiar enough with modelling to run and understand codes like this.

Reviewer #3 (Remarks to the Author):

Hyman et al. present a prospective analysis of potential conflicts between Nickel mining and priority conservation areas, including both terrestrial and deep-sea mining. I greatly enjoyed the paper, which is very clearly written and among the first analyses to compare potential impacts on biodiversity from terrestrial and deep-sea sources, even if in a simplified overlay. I have identified below a number of issues that I hope will help strengthen the paper before acceptance.

Response 3.1: We thank the reviewer for their thoughtful and constructive feedback. We are happy to hear that they enjoyed reading the paper, and we appreciate the recognition of its novelty. We have incorporated all the reviewer's suggestions in our revised manuscript. Please see our responses below.

Main comments

After a mine closes, impacts can vary significantly depending on the country and type of mine. Moreover, once a deposit is fully exploited, the lasting impacts on biodiversity will differ greatly between terrestrial and marine mines. I suggest the authors expand the paragraph starting at L141 to include a more in-depth discussion of their findings in the context of post-mining impacts across different ecosystems.

Response 3.2: Agreed. We have now added the following dedicated paragraph following the discussion of post-mining threats to terrestrial and marine priorities (rather than L141, as this section focuses on supply distribution).

> "Mining activities affect terrestrial, freshwater, and marine biodiversity through numerous impact pathways and mechanisms, with aquatic pollution identified as a pervasive pressure[38]. Strip mining in tropical forests exposes bare ground and waste rock to erosion (Fig. 2), and heavy rainfall mobilises metal-contaminated sediments that can travel tens to hundreds of kilometres downstream, persisting in freshwater systems for centuries or polluting nearshore marine environments[36,37,39]. While the absence of a globally consistent conservation priority layer precluded modelling of threats to freshwater biodiversity, recent assessments show that metal mining is degrading riverine ecosystems worldwide through increased sediment loads and long-distance contaminant transport derived from active and inactive mine sites[36], in addition to artisanal and small-scale operations[40]. In Indonesia, current regulations do not require restoration to pre-mining conditions – emphasising alternative post-mining land uses – and progressive rehabilitation remains rare, despite ongoing deforestation within mining concessions[41]. Without effective rehabilitation, cross-realm threats can persist after mining operations cease, particularly when sites are abandoned. Even with active restoration, forest degradation and biomass carbon loss can often only be partially reversed over reasonable timescales[42,43]. Given the potential growth of nickel mining in highly biodiverse coastal ecosystems in the tropics, subnational assessments of cross-realm threats are a critical research priority."

Similarly, we have also added the following on post-mining impacts of deep-sea mining of polymetallic nodules in the section on the implications of marine resource development:

> "The abyssal seafloor of the CCZ (~4–6 km depth) is a food-limited, low-biomass environment hosting more than 6,000–8,000 metazoan species – primarily small worms and crustaceans from the phyla Annelida, Nematoda, and Arthropoda[23,46]. The lasting biodiversity impacts of deep-sea mining depend largely on the technology used. Modern systems disturb the top 3–5 cm of sediment, whereas long-term disturbance studies focus on systems that were more invasive, such as the Ocean Minerals Company (OMCO) mining test in 1979, which created 20–80 cm deep furrows[23,47–49]. The OMCO test caused long-lasting impacts on invertebrate communities living on the seafloor, while sediment-dwelling organisms and microbial communities had similar densities to undisturbed control sites[48]. Current evidence suggests impacts on nodule-dependent taxa are expected to be long-lasting across contract areas, and recovery will depend on whether there is sufficient hard substrate left unmined to support recolonisation[23,48]. Two months following a recent industrial trial in 2022, sediment-dwelling macrofauna were approximately two-thirds of their baseline density and diversity in directly mined areas[47]. In nearby areas affected by sediment plumes, neither short-term nor long-term studies found significant decreases in faunal abundance compared to control sites[47,48]. We stress that any future polymetallic nodule mining requires effective and evidence-based environmental management to mitigate adverse effects on deep-sea biodiversity and ecosystem services[48,50], and that achieving such outcomes may present a significant challenge[51,52]. We also note that while the development of deep-sea projects may supplement a portion of supply from terrestrial sources, this does not guarantee a wholesale reduction in terrestrial impacts, as existing operators may still fail to scale back land clearing efforts, manage downstream pollution, and implement post-mine rehabilitation."

While I appreciated the detailed discussion on the limitations of the PEMMSS approach, I missed context regarding the limitations of the biodiversity data from Jung et al. and how these limitations influence the results presented in this manuscript. I encourage the authors to discuss potential biases and the representativeness of the dataset used to identify priority conservation areas. Additionally, it would be helpful to clarify how these factors relate to existing protected areas and other commonly used metrics, such as threat status, and to what extent freshwater ecosystems are represented by these layers.

Response 3.3: We thank the reviewer for raising this important point. Please refer to this revised sentence in our methods:

> "We note that although both terrestrial and marine priority maps have coarse resolution, they use the best available data on species distribution, with particular attention to biases introduced by incomplete knowledge on distribution of species in selected global regions (the so-called Wallacean shortfall) and are therefore considered fit-for-purpose for global-scale analyses."

We have also now expanded our discussion of the strengths and limitations of the Jung et al. (2021) priority maps in the "Model limitations":

> "The terrestrial conservation priority maps used in our analysis represent the most comprehensive global integration of biodiversity and carbon data to date, including all extant terrestrial vertebrates (5,685 mammals, 6,660 amphibians, 10,953 birds, and 10,585 reptiles), a representative subset of all accepted vascular plant species (~41%, 193,954 species), and the best available maps of above-ground and below-ground biomass carbon and vulnerable soil carbon[17]. However, the study notably lacks freshwater, soil, and invertebrate species, for which data are currently insufficient for global-scale analyses. Similarly, the ocean priority maps represent the most comprehensive global assessment of priorities for conserving marine biodiversity, integrating multiple data sources[28]. While each study employs realm-specific methodologies for its global optimisation, both consider species threat status, integrate existing protected areas, and conduct extensive uncertainty analyses[17,28]. Although any one analysis has its limitations, multiple global-scale studies confirm that the land and coastal ecosystems of top nickel producers – including Indonesia (Wallacea), the Philippines, and New Caledonia – are global hotspots of terrestrial and marine species endemism[68–70]. We consider the maps used in our analysis appropriate for global-scale analyses of mining threats, but emphasise that focused subnational assessments would be required to evaluate impacts on individual species and ecosystems."

Many of the marine impacts rely on the assumption that mines within 50 km of coastlines contribute to marine impacts. First, for land mines, the radius used to identify overlap with conservation priority areas is 1 to 10 km, which is inconsistent with the 50 km radius. Second, what is the rationale behind the impact mechanisms for land or coastal mines affecting marine biodiversity? It is unclear how the link to the 50 km radius of indirect impacts from Sonter et al. (which remains, in any case, very arbitrary and unverified) indicates how a mine connects land to the coast. I encourage the authors to better support this assumption by explaining the impact pathways that justify using a 50 km radius for land-to-marine impacts. If pollution is the main concern, they could consider using drainage lines with a justified distance threshold (since not all mines within 50 km of the coast might connect to it, depending on local terrain). If the authors decide to maintain a buffer approach, I recommend at least conducting a sensitivity analysis using 10 km and 50 km, as in Sonter et al. (2020).

Response 3.4: We thank the reviewer for these thoughtful comments on our methodological choices regarding the 50 km buffer threshold, which we address in detail below. Our 50 km threshold is justified on multiple grounds and represents a conservative choice relative to documented direct and indirect mining impacts in global-scale analyses. First, our 50 km buffer is constrained by the spatial resolution of the underlying marine conservation priority layer from Sala et al. (2021), which uses ~50 km grid cells (the best resolution available). A 50 km buffer guarantees that at least one raster cell is sampled. Although the ~50 km resolution of the marine priority data precludes a 10 km sensitivity analysis, we have now added Extended Data Fig. 7d to characterise the spatial distribution of deposits relative to coastlines. This figure shows that an estimated ~170 Mt Ni occurs within 50 km of coastlines (~88 Mt Ni within 10 km; ~82 Mt Ni

between 10–50 km), compared to ~68 Mt Ni beyond 50 km. Adopting a 10 km threshold would reclassify ~82 Mt Ni as posing no threat to marine biodiversity, despite evidence that mining impacts extend well beyond 10 km. Given the global scale of our analysis and that most coastal waters adjacent to laterite deposits within 50 km of coastlines rank in the top 10% of marine biodiversity priorities, we consider the 50 km threshold appropriately conservative. We believe the burden of evidence lies with demonstrating that indirect and downstream effects of laterite mining can be contained within 10 km – evidence that does not currently exist. The diverse pathways by which mining activities impact biodiversity is emerging as a critical area of research (see Barbarossa et al.[38], which is cited in our revised manuscript as per Response 3.2). We emphasise that subnational assessments are critical for evaluating mine-specific impact pathways.

While drainage-line analysis would provide finer spatial precision, implementing this approach at a global scale would introduce substantial methodological complexity without materially altering our core findings, given that most laterite deposits occur on small tropical islands where catchments drain directly to the coast. As shown in our new figure (Extended Data Fig. 7d), a significant portion of nickel in known laterite deposits is within kilometers of coastal ecosystems (i.e. often on steep islands in the Coral Triangle). We have added the following sentence to the main text:

> "An estimated 170 Mt of nickel contained in laterite deposits occurs within 50 km of coastlines (88 Mt Ni within 10 km; 82 Mt Ni between 10–50 km), compared to just 2 Mt for magmatic sulphide deposits, which are predominantly located greater than 50 km inland (Extended Data Fig. 7d)."

Where mines drain to closed basins rather than coastal waters, the focus should shift to freshwater contamination – a point we have noted in our revised discussion of cross-realm threats.

We have added the following justification to the Model Limitations section, drawing on literature documenting direct and indirect mining impacts:

> "While significant progress has been made in recent years to map the direct footprint of global mining[56], substantial uncertainty remains regarding indirect and offsite impacts. Available evidence suggests mining poses threats to ecosystems well beyond site boundaries[38,41]. For example, Sonter et al.[10] show that mining-induced deforestation in the Brazilian Amazon can extend up to 70 km from lease boundaries, and Macklin et al.[36] highlight that sediment-associated contaminants can travel 10–100 km downstream. Nickel laterite operations are often expansive and require extensive coastal infrastructure that directly affects local marine environments, exemplified by the construction of major coastal industrial parks in Indonesia in the past decade[15]. In our study, we applied a 50 km buffer to identify deposits with the potential to threaten marine conservation priorities (Fig. 3; Extended Data Fig. 7; Extended Data Fig. 8). This threshold is constrained by the ~50 km resolution of available marine priority data[28], ensuring at least one raster cell is sampled, but is nonetheless consistent with buffers used in recent global mining threat assessments[10]. While global mine-by-mine depletion

> is explicitly represented by our supply scenarios, we do not model the persistence or reduction of threats to conservation priorities following mine closure. Ultimately, global prioritisation studies such as ours should be used to identify areas where regional and local impact assessments may be particularly effective for informing conservation action."

With permission from the photographer Tom Hegen, we have also added a new main text figure (Fig. 2 – adjusting subsequent figure numbers), depicting a coastal laterite mining operation in Sulawesi, Indonesia, with sediment-laden water near a small village, providing a useful visual reference for readers.

Minor comments

L158: Depending on the screen, the 100% shade is barely visible. Please increase the contrast or use contours. The same applies to Figure 2.

Response 3.5: The outer shade represents 100% of simulations, i.e. the top 5% and bottom 5% are not covered by the darker shade. We agree that it can be difficult to see and may make it harder to interpret the graphs. We have decided to remove the outer shade, as we did not deem it necessary for reporting results (which we primarily report 5th to 95th percentiles throughout). This also ensures consistency between line and bar charts. We have updated all associated figures.

L152-163: It is unclear what the "-T" represents in the scenario acronyms just by looking at the figure and caption. I need to get to the Supplement to learn that it refers to terrestrial. Please spell it out in the caption.

Response 3.6: We have updated the captions to explain that -T means terrestrial only scenario at each relevant caption location:

> "The line charts (a and b) show the median annual terrestrial nickel production under the Announced Pledges Scenario (APS-T, where T denotes terrestrial resources only) across model simulations..."

L141-143: How did the authors handle mine closure in their impact assessment modeling? If a mine closes, does the impact continue? In that case, is the mine still included in the projections as a high or low priority with an output of 0 Mt? I suggest adding a sentence explicitly stating whether closure is considered in the modeling and what it entails for the overlay with priority areas.

Response 3.7: Our modelling considers a mine that is depleted as still poseing a threat to biodiversity throughout our 25 year window. Please note, we do not consider our assessment as an "impact assessment", but rather a global threat assessment based on the proximity of deposits to conservation priorities. We have added a sentence to emphasise that our global analysis can directly inform where impact assessments should be prioritised:

> "While global mine-by-mine depletion is explicitly represented by our supply scenarios, we do not model the persistence or reduction of threats to conservation priorities

> following mine closure. Ultimately, global prioritisation studies such as ours should be used to identify areas where regional and local impact assessments may be particularly effective for informing conservation action."

S&P data on ore and concentrate production tonnages is very incomplete, which I assume are crucial for estimating remaining reserves and their grade at existing mines. It would be helpful to have a clear overview of the quality of the S&P data used as input to PEMMSS and to flag the various projects accordingly (low, medium, high quality data).

Response 3.8: We do not use ore or concentrate production tonnages from S&P, as these data are indeed very incomplete. Instead, we use the most recent reported remaining resources (including reserves) for existing mines (and undeveloped deposits) as inputs to the PEMMSS model, where we then estimate production capacity using Taylor's rule (see Methods and Extended Data Fig. 3). This approach avoids reliance on incomplete production data and instead leverages resource estimates that are more consistently reported. We do note the limitations of S&P in our study, while acknowledging it as the best available dataset. For example:

> "However, currently half of global mining areas lack basic production information necessary for independent impact assessments. This is particularly prevalent in Russia, China, Indonesia, and Brazil[56]…"

> "The S&P Database may contain incomplete data, particularly relating to production, due to issues of transparency affecting large portions of the mining industry..."

We have also now added the following sentences to the Model limitations section:

> "Resource reporting quality varies by jurisdiction[56], and established mining regions may have well-documented resources that are unlikely to be developed due to factors beyond the scope of our model (e.g., permitting constraints, community opposition, or insufficient processing capacity), potentially leading to overestimates of production. Conversely, emerging frontiers such as Indonesia may have limited or delayed reporting that does not capture ongoing exploration, potentially leading to underestimates of production."

Ranking the quality of available data would be difficult to do in an objective way, as reporting standards vary by jurisdiction and over time. We believe our approach of using resource estimates (which are more consistently reported under international standards such as JORC and NI 43-101) rather than production data, combined with transparent acknowledgement of data limitations, provides a robust and consistent foundation for our global-scale analysis.

L375-376: Related to the above, where are the results of the validated resource tonnage data against the data from Mervine et al.? They are not referenced with a figure or table here; I would definitely include that.

Response 3.9: Agreed. We have added a new panel (Extended Data Fig. 2b) comparing contained nickel by country and deposit type in our study with estimates from Mervine et al. (2025), who report data up to 2020. The comparison shows general agreement, with increases

in contained nickel in our dataset reflecting exploration and improved reporting in recent years, particularly in Indonesia, Canada, and New Caledonia.

Reviewer #4 (Remarks to the Author):

This is an excellent manuscript that addresses an important and understudied aspect of moving towards a net-zero future, the adverse biodiversity impacts of a growing nickel supply required to power a net-zero future. The authors explore this through the new PEMMSS model, a model that is firmly gounded in reliable and sound data sources. By combining the IEA's global decarbonization scenarios with global conservation targets the modeling assumptions are reasonable and rooted in the latest research and policy discussions. The manuscript is also important by highlighting Indonesia's outsized role in the current nickel supply, a recent phenomenon with massive adverse environmental and biodiversity impacts. The methodology is sound, includes reasonable and relevant scenarios, and good sensitivity analyses. The authors may want to explain a bit more why the possibility to supply nickel from secondary (recycled) sources is so limited. The one sentence provided (line 115) may not be easily understood by readers that don't follow critical material discussions closely. This could include a reminder of the importance of recycling (and circular economy, design for recycling). With this, I consider the manuscript ready for publication.

Response 4.1: We thank the reviewer for their positive review and recommendation for publication. We appreciate the suggestion to expand our explanation of secondary supply constraints. We have now amended the following paragraph on why secondary supply is limited:

> "Secondary supply (nickel recovered from recycling) will become increasingly important by mid-century to reduce reliance on mined nickel but is projected to play a minor role in the near-term (~2% in 2024, ~1.5–3% by 2030, ~5–10% by 2040 across STEPS and APS scenarios[3,29]), constrained by the rate at which products reach their end of life for recycling[3]. Nickel is predominantly used in long-lived applications, including stainless steel in buildings and infrastructure (~50 year lifespan) and industrial machinery (~25 years)[30,31]. The amount of nickel available for recycling today reflects the lower production volumes of past decades[26], and the in-use stocks being accumulated now may only become available for recycling at significant volumes by mid-century[3,32,30]. Current end-of-life recycling rates fall far short of what is needed to offset primary supply requirements, and scaling secondary supply will require improvements in collection systems, urban mining, and design for recyclability[32,33]. Even with increasing adoption of low- or no-nickel battery chemistries[3,7], total demand is projected to grow substantially across all of the International Energy Agency's scenarios due to nickel's importance for stainless steel, renewables, and other applications (Extended Data Fig. 1)."

Minor comment:
Line 293: I'd suggest slightly rephrasing this sentence, adding the caveat that "current" decarbonization pathways require lots of nickel, while technology change might mitigate this high demand.

Response 4.2: Agreed. We have amended the sentence:

"Current pathways to decarbonisation require a substantial increase in nickel supply over the coming decades, although the pace of demand growth will depend on policy ambitions and technological developments."
